# Paired tumor sequencing and germline testing in breast cancer management: An experience of a single academic center

**DOI:** 10.1002/cnr2.1287

**Published:** 2020-09-03

**Authors:** Elizabeth Elliott, Virginia Speare, James Coggan, Carin Espenschied, Holly LaDuca, Amal F. Yussuf, Kelly Burgess, Phillip Gray, Melody Cobleigh, Ruta Rao, Jeremy Patel, Timothy Kuzel, Lela E. Buckingham, Lydia Usha

**Affiliations:** ^1^ Department of Medicine, Division of Hematology, Oncology, and Stem Cell Transplant Medicine Rush University Medical Center Chicago IL USA; ^2^ Department of Pathology Rush University Medical Center Chicago IL; ^3^ Ambry Genetics Aliso Viejo California USA

**Keywords:** breast cancer, cancer predisposition, clinical utility, genomic profiling, germline testing, next‐generation sequencing

## Abstract

**Background:**

Genetic testing for cancer predisposition is recommended to women with breast cancer who meet the criteria for such testing. After the FDA approvals of the poly ADP ribose polymerase (PARP) inhibitors, olaparib and talazoparib, for treatment of metastatic breast cancer, carrying germline mutations in *BRCA1* and *BRCA*2 genes, the genetic testing result has become critical in their care. With the recent FDA approval of alpelisib for the treatment of *PIK3CA*‐mutated hormone‐receptor positive metastatic breast cancer, tumor molecular profiling to identify somatic mutations and potential molecularly targeted agents is increasingly utilized in the treatment of advanced breast cancer.

**Aim:**

Combining germline and somatic sequencing (paired testing) offers an advantage over a single technique approach. Our study evaluates the role of paired testing on the management of breast cancer patients.

**Methods and Results:**

Forty‐three breast cancer patients treated at Rush University Medical Center underwent paired germline and somatic variant testing in 2015 to 2017. A retrospective chart review was conducted with the analysis of demographic, clinical, and genomic data. Three actionable germline variants were found in the *CHEK2* (2) and *ATM* (1) genes. 95% of tumors had somatic mutations. Seventy‐seven percent of tumors had genomic alterations targetable with agents approved for breast cancer and 88% had molecular targets for agents approved for other cancers. Clinical examples of such use are described and potential future directions of tumor and paired testing are discussed.

**Conclusions:**

Germline variants were present in a relatively small patient group not routinely tested for inherited alterations. Potentially targetable somatic alterations were identified in the majority of breast cancers. Paired testing is a feasible and efficient approach that delivers valuable information for the care of breast cancer patients and eliminates serial testing.

## 1 INTRODUCTION

Recent advances in precision oncology allow for the administration of more effective and less toxic treatments, leading to improved patient outcomes. Molecularly targeted agents (MTAs), such as imatinib in chronic myelogenous leukemia or trastuzumab in Her‐2/neu‐positive breast cancer, have shown significant prolongation of overall survival in the corresponding malignancies and sometimes, cures.^1^ A meta‐analysis of Phase I trials with more than 13 000 patients showed that biomarker‐driven MTAs as compared to non‐personalized treatments resulted in higher response rates (30.6% vs 4.9%) and better progression‐free survival (PFS; 5.7 months vs 2.9 months).^2^ In addition, MTAs usually have fewer side effects (on‐target or off‐target) than cytotoxic agents. There is an ever‐increasing number of clinical trials underway, testing new targets and matching therapies.^3^ In January of 2018, a PARP‐inhibitor, olaparib, became the first genomically based treatment approved by the United States FDA (Food and Drug Administration) for patients with metastatic breast cancer (MBC), specifically those who carry germline *BRCA1* or *BRCA2* mutations.^4^, ^5^ Subsequently, two more MTAs (talazoparib and alpelisib) have been FDA‐approved for metastatic breast cancer with a germline *BRCA* mutation (talazoparib) and somatic *PIK3CA* alteration (alpelisib).^6^, ^7^ Advances such as these confirm the promise of molecularly based approaches in breast cancer (BC) management. Currently, therapy in BC is guided by the presence of estrogen and progesterone receptors as well as human epidermal growth factor receptor 2 (HER2) protein overexpression or amplification on pathologic examination.^8^, ^9^, ^10^, ^11^ The introduction of next‐generation DNA sequencing (NGS) and recently, mapping, whole cancer genomes to find cancer “drivers,” allowed to identify promising MTAs.^12^, ^13^ At present, however, alpelisib is the only MTA that requires identification of *PIK3CA* mutation in the tumor to predict its efficacy. Tumor molecular profiling (TMP) is usually employed in advanced BC when all standard treatments have been exhausted.^14^ Since the technology of TMP with NGS became available, many experts considered it investigational and appropriate only in a clinical trial setting. Yet, in March of 2018, Centers for Medicare & Medicaid Services made the decision that NGS is “reasonable and necessary” in advanced cancer for Medicare beneficiaries, opening the doors for wider use of this laboratory testing.^15^ NGS is also employed in germline hereditary predisposition testing, which typically involves the use of multi‐gene panels on constitutional DNA isolated from blood cells. Germline testing provides valuable information on cancer surveillance and risk reduction for patients and their biological relatives. It may also provide information for MTA selection, such as identifying a germline *BRCA1/2* mutation in a patient with metastatic BC who may benefit from treatment with poly ADP ribose polymerase (PARP) inhibitors. Combining germline testing with TMP (paired testing or PT) may provide a time and resource efficient option to reveal molecular targets for therapy and genetic predisposition to cancer.^16^

We performed a pilot study of PT in a series of 43 BC patients to assess the feasibility and clinical impact of this testing approach at a single academic medical center. We discuss the implications of PT results for treatment selection, subsequent cancer surveillance, and screening of family members. We also discuss the efficiency of the PT approach.

## 2 METHODS

We performed a retrospective chart review of women with the diagnosis of BC who underwent PT when deemed appropriate by their oncologists in the context of routine clinical care at Rush University Medical Center from November 2015 to February 2017. The following data were collected: patient age, ethnic background, clinical stage at diagnosis, histology, ER/PR/HER2 receptor status, BC treatment history, and PT results. All patients received pre‐test risk assessment and genetic counseling to inform them about PT. Post‐test genetic counseling was provided for patients and/or their families who tested positive for a pathogenic or likely pathogenic germline variant. The study was approved by the Rush University Institutional Review Board (IRB# 17041703‐IRB01). Participants were de‐identified before group statistical analyses.

### 2.1 Laboratory analysis

DNA was extracted from a primary tumor for the majority of subjects while a metastatic lesion was analyzed when the primary tumor was not available. Blood or saliva was also submitted for each patient for germline DNA extraction. Samples were processed as previously described.^16^ PT consisted of a custom probe‐based NGS tumor panel (Illumina HiSeq) for the detection of single nucleotide variants, small insertions, and deletions in 142 genes that frequently harbor somatic and/or germline mutations in cancer (Online Resource Table S1). The NGS panel used detects gene fusions and structural variants, such as tandem duplications and inversions, in 15 frequently disrupted oncogenes and tumor suppressors. Tumor tissue and a matched blood specimen were analyzed using a custom bioinformatics pipeline to differentiate between somatic and germline mutations, allowing for precise variant classification. Briefly, paired normal samples were analyzed using Novoalign V3.02.07 to align FASTQ reads to a reference sequence (hg19) and GATK (V3.2.2) to generate variants and no/low coverage reports. Germline variants were filtered using a Q score of 30, coverage of 10X, het ratio of 10%, and filtered out if determined to be a sequencing artifact or common polymorphism, utilizing population frequency data from NCBI dbSNP, NHLBI Exome Sequencing Project (ESP), 1000 Genomes, and internal Ambry data. In tumor‐normal analysis mode, Varscan2 (v2.3.6) was used to detect somatic variants as low as 3% minor allele frequency. Structural variants were annotated using Oncofuse v1.0.7 and DELLY v.0.6.1, respectively. Germline genetic variants were assessed using Ambry's five‐tier classification framework based on guidelines published by the American College of Medical Genetics and Genomics and the Association for Molecular Pathology (pathogenic; likely pathogenic; variant of uncertain significance; likely benign; benign).^17^, ^18^ Tumor specimens were also analyzed using the Affymetrix OncoScan platform, an array technology for high‐resolution copy number variant detection that can detect single copy amplifications, hemizygous deletions, and copy neutral loss of heterozygosity.

Alterations identified in TMP and associations with MTAs were reported based on peer‐reviewed studies and other publicly available resources. Germline variants when identified in blood or tumor were reported for the following genes: *APC*, *BRCA1*, *BRAC2*, *MLH1*, *MSH2*, *MSH6*, *MUTYH*, *PMS2*, *PTEN*, *RB1*, *RET*, *SDHAF2*, *SDHB*, *SDHC*, *SDHD*, *STK11*, *TP53*, *TSC1*, *TSC2*, and *VHL*. In some cases, germline testing was performed on additional cancer susceptibility genes as clinically indicated based on genetic risk assessment. Actionable germline alterations were defined as those alterations associated with a currently available option for cancer surveillance, prevention, or treatment in either the patient or their close family members.

## 3 RESULTS

Age at diagnosis, ethnicity, stage at the time of testing, histologic type, and tumor markers of breast cancer for 43 patients are reported in Table 1. Our patient population had a greater proportion of stage IV disease at diagnosis and a younger age than average BC population when compared to National Cancer Institute (NCI) epidemiological data, which may reflect the referral bias since patients with advanced disease were more likely to get referred for paired testing.^19^, ^20^ The primary histological BC subtype of this patient group was infiltrating ductal carcinoma (IDC), which correlates with NCI data, yet there was a somewhat larger proportion of infiltrating lobular carcinoma, which may be the result of a small sample.^19^ The majority of our patients had hormone receptor positive, HER2 negative BC, consistent with national data.^21^

**TABLE 1:** Demographic and clinical characteristics of breast cancer cases

Patient characteristics		Patients with MTA for breast cancer (%)
Number of patients	43	33/43 (76.7)
Median age at diagnosis (IQR^a^) [years]	66 (53, 72)	‐
Median age at tumor testing (IQR) [years]	64 (51, 70.5)	‐
Age at diagnosis group	N (%)	
30‐39	1 (2.3)	1/1 (100.0)
40‐49	7 (16.3)	2/7 (28.6)
50‐59	10 (23.3)	8/10 (80.0)
60‐69	12 (27.9)	9/12 (75.0)
70‐79	6 (14.0)	6/6 (100.0)
80 and over	7 (16.3)	6/7 (85.7)
Race/ethnicity (%)		
Caucasian	23 (53.4)	19/23 (82.6)
Ashkenazi Jewish	0 (0.0)	‐
African American	12 (27.9)	9/12 (75.0)
Asian	1 (2.3)	0/1 (0)
Hispanic	4 (9.3)	4/4 (100.0)
Multiple/other/unknown	3 (7.0)	1/3 (33.3)
Clinical stage at time of paired testing (%)		
I	4 (9.3)	3/4 (75)
II	6 (14.0)	5/6 (83.3)
III	4 (9.3)	2/4 (50.0)
IV	23 (53.4)	20/23 (87.0)
Incomplete early stage^b^	6 (14.0)	3/6 (50.0)
Histology		
IDC	29 (67.4)	26/29 (90.0)
ILC	7 (16.3)	5/7 (71.4)
Mixed histology	6 (14.0)	1/6 (16.7)
Unknown histology	1 (2.3)	1/1 (100.0)
Receptor status		
Triple negative (ER‐/PR‐/Her2‐)	5 (11.6)	2/5 (40.0)
(ER+/PR+/Her2+) or (ER+/PR‐/Her2+) or (ER‐/PR+/Her2+)	6 (14.0)	3/6 (50.0)
(ER+/PR+/Her2‐) or (ER+/PR‐/Her‐2‐) or (ER‐/PR+/Her2‐)	28 (65.1)	26/28 (93.0)
(ER‐/PR‐/Her2+)	4 (9.3)	4/4 (100.0)

*Note*: This table depicts demographic and clinical characteristics of the breast cancer cases included in this study.

^a^ IQR = interquartile range.

^b^ T1 − T2 tumors without nodal assessment.

Somatic genomic alterations in the tumors tested were highly prevalent with 95% (42/43) of tumors having at least one reported mutation with an average of 6.5 (range 0‐17) mutations per tumor. Overall, 77% of tumors contained at least one target for BC therapy, and 88% contained an FDA‐approved targeted therapy for another type of cancer, potentially available off‐label for BC. (Table 2).

**TABLE 2:** Genomic alterations and available therapy and clinical trials

Genomic alterations (patients)^a^	278 (43)
Patients with at least one FDA approved therapy for breast cancer (% of tumors affected)	33 (76.7)
Patients with at least one FDA approved therapy for other tumor type (% of tumors affected)	38 (88.3)
Patients who were eligible for a clinical trial based on genomic alteration(s) (% of tumors affected)	40 (93.2)

*Note*: This table depicts clinical implications of genomic alterations identified. It stratifies results based on percentage of total alterations meeting specific clinical criteria, and percentage of tumors included in the study, which meet clinical criteria mentioned.

^a^ Gene alterations found in multiple patients were counted separately.

**FIGURE 1:**
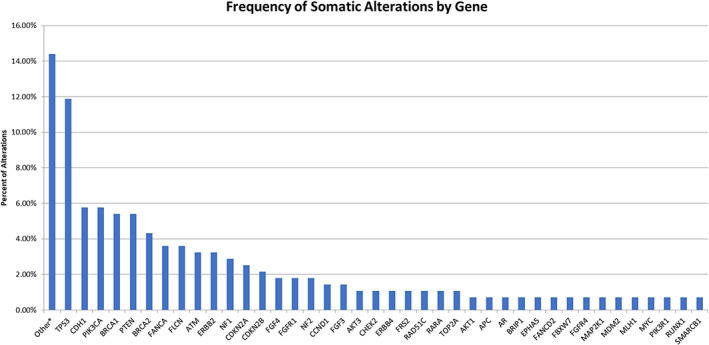
Frequency of somatic alterations by gene. This figure depicts frequency of specific genomic mutations identified across all tumors studied. Two hundred and seventy‐eight somatic alterations (mutations, allelic loss, and amplifications) in 81 genes were clinically reported. The single most frequently observed gene was *TP53*. Genes known to be frequently mutated in BC (*BRCA1*, *BRCA2*, *CDH1*, *PIK3CA*, *PTEN*, including *TP53*) accounted for 40% of alterations

**FIGURE 2:**
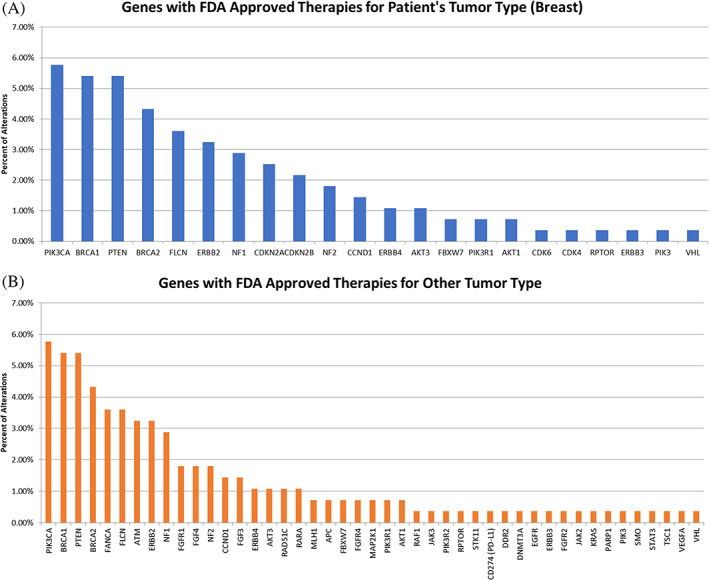
Genes with Associated FDA Approved Therapy. This figure depicts frequency of genomic mutations with FDA approved therapies (both for breast cancer and for other tumor types) identified across all tumors studied. Twenty‐two genes altered in this patient group had an associated MTA for breast cancer (A). Alterations in additional genes had an associated MTA for other cancers (B)

A total of 278 alterations in 81 genes were clinically reported in Figure 1, and are broken down by ER receptor status in Graph S1. Hemizygous loss of a gene was the most common alteration (n = 96) while missense alterations (n = 79) and gene amplifications (n = 66) were also frequent (Online Resource Table S2). Genes known to be frequently mutated in BC (*BRCA1*, *BRCA2*, *CDH1*, *PIK3CA*, *PTEN*, and *TP53*) accounted for 40% of alterations. Twenty‐two genes, altered in this cohort of patients, had an associated MTA for breast cancer while alterations in additional genes added to the number of patients who were potentially eligible for an MTA (Figure 2). Patient characteristics in relation to the alterations can be found in Online Resource Table S3.

Three patients were found to have actionable germline variants that were not previously suspected including one pathogenic mutation in *CHEK2* (c.1100delC), a likely pathogenic variant in the *CHEK2* gene (c.1427C > T), and a likely pathogenic splice site variant in the *ATM* gene (2466 + 1G > C). None of these three patients met the then current National Comprehensive Cancer Network (NCCN) germline genetic testing criteria.^22^ We have performed TMP on breast cancers of the three germline carriers described above (Table 3). Two of these tumors had multiple molecular alterations.

**TABLE 3:** Somatic variants in tumors of three identified germline carriers

Patient	Germline mutation	Somatic variants	Selected demographic and clinical characteristics. Disease Status as of July 2020
1	*CHEK2*, likely pathogenic c.1427C > T (p.Thr476Met)	Four somatic single nucleotide variants (SNVS): *PIK3CA* p. H1047L *AR* p.R13W *TP53*, p. Y220C *CDH1* p.N297S	Caucasian Diagnosed in 2011 at age 64 with stage I breast cancer ER and PR positive, Her‐2/neu negative. Currently, NED Also, has a history of clear cell papillary renal cell carcinoma
2	*ATM*, likely pathogenic c.2466 + 1G > C (splicing site exon 18)	10 somatic alterations: *CDKN2A* hemizygous loss *CDKN2B* hemizygous loss *PTEN* hemizygous loss *NF2* hemizygous loss *BRCA2* hemizygous loss *ATM* LOH *FANCA* hemizygous loss *FLCN* hemizygous loss *T53* (pT155_R156del and T53 LOH)	Caucasian Diagnosed in 2015 at age 50 with stage II breast cancer, ER positive, PR and Her‐2/neu negative. Currently, NED.
3	*CHEK2*, pathogenic c.1100del (pT367Mfs*15)	13 somatic alterations: *CDKN2A* hemizygous loss *CDKN2B* hemizygous loss *PTEN* hemizygous loss *NF1* hemizygous loss *BRCA1* hemizygous loss *ATM* hemizygous loss *FLCN* hemizygous loss *T53* hemizygous loss *CDH1* hemizygous loss *TSC1* hemizygous loss *APC* hemizygous loss *CDK4* amplification *FRS2* amplification	African‐American Diagnosed in 2016 at age 79 with stage II breast cancer, ER positive, PR and Her‐2/neu negative Died in 2019 with no evidence of breast cancer recurrence.

*Note*: This table captures somatic variants and selected clinical and demographic information for the three patients with germline variants that were not previously suspected.

### 3.1 Clinical application of PT results

Because 47% of our sample (20 patients) consisted of patients with early stage breast cancer, real‐time clinical care was usually not impacted by PT but rather, adjuvant or neoadjuvant treatment was based on stage, pathologic characteristics of the tumor including tumor markers, and, in some cases, on the results of molecular prognostic assays such as Oncotype DX and Mammaprint. In one patient (Patient C), an MTA was considered in adjuvant setting and in two women (Patient A and Patient B) MTAs were administered when BC recurred.

Patient 404 122 (Table S3) was diagnosed with inflammatory triple‐negative left‐sided IDC, clinical stage III, grade 3, at age 66. She received four cycles of neoadjuvant dose‐dense doxorubicin and cyclophosphamide, followed by four cycles of paclitaxel, and underwent left modified radical mastectomy, which demonstrated significant residual BC, ypTis N3a. After radiation to the left chest wall, adjuvant chemotherapy with carboplatin was attempted but not tolerated due to rash and weakness. She was diagnosed with histologically confirmed recurrence of triple‐negative BC and metastases to intra‐thoracic lymph nodes a year after her surgery. PT was performed on the primary tumor and revealed four somatic genomic alterations including *KRAS* amplification, *BARD1* p.A724V, *TP53* p. K139_P142del, and *TP53* copy neutral LOH. Based on these findings, she was prescribed a suggested MTA off‐label, Trametinib, a mitogen‐activated protein kinase (MEK) inhibitor, which targets *KRAS* amplification. Although it does not directly inhibit MEK, trametinib has been shown to limit tumor progression via CD8 T‐cell mediated factors by altering signaling along the RAS‐ERK pathway in cancers with *KRAS* amplification.^23^ Unfortunately, trametinib was discontinued 1 month later due to progression.

Patient 435 327 (Table S3) was diagnosed with clinical stage III, grade 3, triple‐negative right‐sided IDC at age 62. She received four cycles of neoadjuvant dose‐dense doxorubicin and cyclophosphamide, followed by 12 cycles of weekly paclitaxel. She underwent subsequent right modified radical mastectomy with residual tumor noted, ypT2N0. She then completed radiation and received six cycles of adjuvant chemotherapy with capecitabine. She also was diagnosed with BC recurrence and histologically confirmed pulmonary metastases 1 year after surgery. She underwent PT using a tissue sample from her mastectomy, notable for four somatic genomic alterations: *PIK3R1* p.R461_E462delinsQ, *FGF3* amplification, *FGF4* amplification, and HER2‐Neu (*ERBB2*) mutation, p. S310F. Given the finding of *HER2‐Neu* mutation, she was offered participation in phase II study with neratinib.^24^ Because somatic *HER2‐Neu* mutations activate the *HER2‐Neu* oncogene without its amplification, Trastuzumab and other commonly used *HER2‐Neu* directed agents are usually ineffective in *HER2‐Neu*‐mutated BC. Neratinib is a small molecule, which irreversibly inhibits both HER2‐Neu amplifications and mutations.^25^ The patient enrolled in this trial but it was discontinued 3 months later due to cancer progression.

Patient 487 820 (Table S3) was diagnosed with clinical stage III, Grade 2, ER‐positive, PR‐negative, HER2‐negative IDC with neuroendocrine features at age 52. She received neoadjuvant doxorubicin and cyclophosphamide, followed by dose‐dense paclitaxel. She underwent total left mastectomy with sentinel lymph node biopsy, which showed residual BC, ypT1cN1. PT of her surgical sample revealed somatic mutations in *TP53* and a hemizygous loss of *NF2*, yielding four possible therapies, including an mTOR inhibitor, everolimus, approved in combination with anti‐hormonal therapy for metastatic BC. Given the significant amount of residual disease and this molecular alteration, she was evaluated for the adjuvant trial with everolimus.^26^ However, she was found to be ineligible because positive lymph node biopsy was not followed by axillary lymph node dissection based on patient and surgeon preference. She underwent adjuvant radiation therapy and started anti‐hormonal therapy with tamoxifen. At the time of this writing, she remains without evidence of disease 3.5 years after surgery.

## 4 DISCUSSION

In our study, 95% of all tumors analyzed demonstrated at least one somatic mutation presumed to be involved in tumorigenesis. The most commonly detected mutation was in *TP53* gene, followed by *CDH1* and *PIK3CA*. This correlates with other studies, which have described *TP53* as the most commonly mutated gene in human cancers.^27^ As seen in Figure 1, the rate of gene alteration in *TP53* was less than 12% and that in *PIK3CA* was less than 6%, which are lower than reported results for breast cancer. Those studies, however, include non‐coding regions with high mutation frequencies, not all of which can be classified as driver mutations. Mutational signatures extended to genome rearrangements, characterized by tandem duplications or deletions, appear to be associated with defective homologous recombination‐based DNA repair.^28^ The analysis of all classes of somatic mutation across exons, introns, and intergenic regions might generate a higher overall mutation rate, not all of which will be driver mutations. In general, mutations in tumor suppressor genes (loss of function mutations) like *TP53* are more difficult to target than mutations in oncogenes (gain of function mutations).^29^ PARP‐inhibitors are the first class of MTA that targets mutations in tumor suppressor genes (*BRCA1* and *BRCA2*). Preliminary data suggest that these agents can also elicit synthetic lethality in the presence of mutations in other tumor suppressor, such as *TP53* and *PTEN*, which are mutated in 37% and 3% of all BCs, respectively, and represented 11.8% and 5.4% of mutations in our sample.^30^, ^31^

Gain‐of‐function mutations in genes such as *PIK3CA*, which allow unregulated cell proliferation, had associated MTAs (mTOR inhibitors: temsirolimus and everolimus; recently approved *PIK3CA* inhibitor: alpelisib) while others, such as a loss‐of‐function mutations in the tumor suppressor gene, *CDH1*, were not targetable in our patients (Table S2). Notably, only one of our tumor samples demonstrated a mutation of *ESR1* (estrogen receptor 1). This may be explained by the number of primary, non‐metastatic breast tumor tissue in our sample, as the incidence of *ESR1* mutations rises to 15% to 20% in metastatic ER‐positive tumors after prior endocrine treatments, suggesting the development of resistance.^32^

A molecular target for breast cancer treatment was available for a majority (33/43) of patients. However, we did not observe responses in two patients who received MTAs. This is consistent with the initial results of clinical trials looking at the use of MTAs in prospective fashion.^33^, ^34^ For instance, in SAPHIR01 trial, for women with metastatic BC, 13% of patients were able to receive MTAs based on the genomic data and of those patients, only 9% had objective tumor response.^35^

When analyzing the results of MTA clinical trials, it is important to remember that they depend on target identification in tumor tissue and availability of matching MTAs. In the initial phase of the NCI sponsored MATCH (Molecular Analysis for Therapy Choice) trial, only 9% of patients had a matched targeted therapy, when there were only 10 MTAs and 10 corresponding arms in the study. However, the percentage of patients increased to 23% when the number of MTAs and the respective arms in the study went up to 24%.^36^

Previous studies have revealed the prevalence of germline variants discovered in paired testing. A retrospective study of Stanford patients with *BRCA1/2* somatic mutations found 55.7% were positive for pathogenic *BRCA1/2* germline mutations, confirming “a second hit” hypothesis.^37^ In a study of patients with advanced cancer diagnoses, presumed pathogenic germline variants were found in 17 of 269 (6.3%) breast cancer patients (6.3%).^38^ In another study of 1040 breast, prostate, renal, pancreatic, and colon cancer patients, 182 (17.5%) had clinically actionable mutations conferring cancer susceptibility. Of these, 101 patients would not have had these mutations detected using clinical guidelines. Germline findings led to predictive testing in the families of 13 individuals (1.3%), including 6 for whom genetic evaluation would not have been initiated by guideline‐based testing.^39^ In our small sample, we identified three patients (7.0% of those tested) with previously unrecognized actionable germline mutations in *CHEK2* and *ATM* genes, which are consistent with these data.

Variants in *CHEK2* and *ATM* can impact cancer surveillance and cancer screening, as well as prevention for blood relatives.^40^, ^41^ For our patients, the discovery of these actionable germline mutations led to recommendations for increased breast cancer surveillance with the addition of screening breast MRIs (for both *ATM* and *CHEK2* mutation carriers) and more frequent colonoscopies for colon cancer surveillance (for *CHEK2* mutation carriers only).^42^ The same recommendations would apply to their blood relatives if they carry these mutations. Currently, germline genetic testing for hereditary predisposition to breast and other cancers is only offered to individuals who meet specific testing criteria set forth by different societal guidelines, most commonly, the National Comprehensive Cancer Network (NCCN) Clinical Practice Guidelines. The NCCN guidelines recommend genetic testing for *BRCA1/2* mutations based on age at breast cancer diagnosis and burden of breast, ovarian, and other related cancers in the family. While these guidelines are useful to identify individuals who should be considered for genetic testing, germline mutations in the *BRCA1/2* and other BC susceptibility genes may be missed in individuals who do not meet these criteria as their sensitivity is limited.^43^, ^44^ PT may allow for detecting pathogenic germline mutations in such BC patients. Importantly, somatic *BRCA 1/2* mutations in the tumors of *BRCA* 1/2 mutation carriers can cause functional reversal of the germline mutation and restoration of the wild‐type BRCA, which results in resistance to carboplatin and PARP‐inhibitors in these patients. As opposed to “a second‐hit” mutation, which affects a normal allele, a “reversing” mutation usually affects the allele with a germline mutation.^45^

Ongoing studies evaluate a potential benefit of PARP‐inhibitors in the presence of somatic *BRCA1/2* mutations as well as germline and somatic mutations in other genes in homologous recombination (HR) pathway, including *ATM* and *CHEK2*. In a Phase 2 study of olaparib monotherapy in metastatic breast cancer patients with germline or somatic mutations in DNA repair genes (Olaparib Expanded), the response to olaparib is studied in patients with metastatic BC and germline or somatic mutations in the following genes: *ATM*, *ATR*, *BARD1*, *BRIP1 (FANCJ)*, *CHEK2*, *FANCA*, *FANCC*, *FANCD2*, *FANCE*, *FANCF*, *FANCM*, *MRE11A*, *NBN*, *PALB2*, *RAD50*, *RAD51C*, *and RAD51D*.^46^ In our cohort of women, if their BC recurs, three germline mutation carriers can be candidates for this trial as well as seven patients with somatic mutations in at least one of the above‐listed genes.

The same genomic alterations can be a target for a chemotherapy agent carboplatin, which is known to double the objective tumor response rate in women with metastatic BC and germline *BRCA1/2* mutations.^47^ HR deficiency in early stage BC as measured by a Homologous Recombination Deficiency (HRD) molecular assay was associated with a higher chance to benefit from carboplatin in neoadjuvant setting.^48^, ^49^ In a study of triple‐negative breast cancer, 59% of cases with homologous‐recombination‐repair deficiency had better outcome on adjuvant chemotherapy for invasive disease‐free survival (hazard ratio 0.42) compared to those without, regardless of whether a genetic/epigenetic cause was identified.^13^

In our study, all three germline mutation carriers had mutations in HR pathway (*CHEK2 and ATM* genes). In addition, the somatic alterations in two of these three tumors also included many genes in HR pathway (*BRCA1 and BRCA2*, *NF1 and NF2*, *FANCA*) (Table 3). It is logical to suggest that these somatic alterations (frequently, hemizygous losses) in the same pathway played a key role in the tumor development initiated by the germline mutation. Given this constellation of germline and somatic variants, it is likely that these cancers would respond to PARP‐inhibitors and carboplatin if they recur.

As discussed earlier, TMP and PT data are usually not incorporated in treatment decisions for early BC. However, this may change with the increased use of platinum agents for adjuvant and neoadjuvant treatment of BC in *BRCA1/2* mutation carriers. Moreover, clinical trials are looking at a potential benefit of PARP‐inhibitors in adjuvant and neoadjuvant treatment of high‐risk BC patients with these mutations.^50^ A recent study showed that patients with BC harboring kinase or helical domain *PIK3CA* mutations derived significantly greater benefit from letrozole over tamoxifen in BIG 1‐98 adjuvant trial.^51^ If confirmed prospectively, this molecular finding can inform the selection of adjuvant anti‐hormonal agents in the future. An integrative analysis of 2658 whole‐cancer genomes and their matching normal tissues across 38 tumor types from the pan‐cancer analysis of whole genomes (PCAWG) consortium found cancer genomes contained 4 to 5 driver mutations, however, in around 5% of cases no drivers were identified, suggesting that cancer driver discovery is not yet complete.^12^

Our study showed that the use of PT is feasible in an actual clinical setting and a single academic institution. Even though TMP can potentially identify germline mutations, their ascertainment as such is difficult without a paired germline testing.^52^ PT is more efficient than a germline testing or tumor sequencing alone, providing valuable information for the care of BC patients and eliminating their “testing fatigue” when asked to do serial tests; however, larger prospective studies are needed to assess the clinical impact and cost‐effectiveness of PT for women with newly diagnosed BC.

## 5 CONCLUSIONS

With the rising demand for targeted therapies and the rapidly changing landscape of MTAs, it is obvious that tumor sequencing holds promise for BC. Outcomes of the three patients in this study, treated with MTA, based on somatic alterations, demonstrate the limitations that remain for their current use. In the years to come, cancer treatment may be dictated more by tumor mutations than tumor type. Yet, before BC tumor sequencing and paired testing become routine, active targets must be validated and more MTAs developed, including those that aim at targets currently deemed “not‐druggable.” Achieving these goals is likely to improve insurance reimbursement for BC sequencing and PT, which, at present, is often lacking.

Clinical trials are desperately needed to evaluate the efficacy and safety of MTAs. However, enrollment into these trials can be challenging as they are increasingly conducted in smaller biomarker‐enriched patient populations selected by the presence of the target. In addition, since <1% to 20% of tumors harbor individually rare somatic mutations, collecting and reporting individual responses to MTAs as described in this paper (“N‐of‐one experiences”) are crucial for future success of MTAs.^53^ For this reason, we support the establishment of an up‐to‐date web‐based open‐access database of molecular targets and responses to MTAs in individual patients, such as the ASCO initiative CancerLinQ.^54^

Analysis of cancer‐related genes in paired germline and tumor DNA samples can lead to increased detection of clinically significant heritable mutations compared to the predicted yield of targeted germline testing based on current clinical guidelines. Identification of germline variants can help guide therapeutic and preventive interventions. Drawing from our experience in this study, we predict expanding indications for paired somatic and germline testing in the future for BC, as well as other types of cancer.

## CONFLICT OF INTEREST

At the time research was conducted, Virginia Speare, Holly LaDuca, Carin Espenschied, Amal Yussuf, and Philip Gray were full time employees of Ambry Genetics. Carin Espenschied is a current employee and stock holder at Guardant Health. Otherwise we have no other conflicts of interest to report.

## AUTHORS' CONTRIBUTIONS

All authors had full access to the data in the study and take responsibility for the integrity of the data and the accuracy of the data analysis. *Conceptualization*, L.U., C.E., PG; *Methodology*, E.E., V.S, K.B., P.G., C.E., L.U.; *Investigation*, E.E., M.C., R.R., K.B., H.L., A.Y., L.U.; *Formal Analysis*, P.G., A.Y., V.S.; *Resources*, M.C., R.R., P.G., L.U; *Writing ‐ Original Draft*, E.E., J.C., J.P., K.B., and L.U.; *Writing ‐ Review & Editing*, E.E., J.C., J.P., K.B., V.S., C.E., H.L., A.Y., P.G., T.K., L.B., H.L., M.C., R.R, and L.U.; *Visualization*, C.E., A.Y., V.S, J.C.; *Supervision*, C.E., L.U., V.S.; *Funding Acquisition*, L.U., H.L., P.G., V.S.

## ETHICAL STATEMENT

The study was approved by the Rush University Institutional Review Board (IRB# 17041703‐IRB01).

## Supporting information


**Data S1.** Supporting information.

## Data Availability

Our data is not currently shared in a repository, but can be made available upon request.
