# Supplementary material for: Paired tumor sequencing and germline testing in breast cancer management: An experience of a single academic center
Source: Cancer Rep (Hoboken). 2020 Sep 3;3(6):e1287. doi: 10.1002/cnr2.1287 (PMC7941483; doi:10.1002/cnr2.1287)
Supplement: Supplementary file 1 — Data S1. Supporting information. [file CNR2-3-e1287-s001.pdf]

WILEY CANCER REPORTS

Paired Tumor Sequencing and Germline Testing in Breast Cancer Management: An Experience of a Single Academic Center

Journal:	*Cancer Reports*
Manuscript ID	CNR2-20-0025.R2
Wiley - Manuscript type:	Original Article
Date Submitted by the Author:	05-Aug-2020
Complete List of Authors:	Elliott, Elizabeth; Rush University Medical Center<br>Speare, Virginia; Ambry Genetics Corp<br>Coggan, James; Rush University Medical Center<br>Espenschied, Carin ; Ambry Genetics Corp<br>LaDuca, Holly; Ambry Genetics Corp<br>Yussuf, Amal; Ambry Genetics Corp<br>Burgess, Kelly; Rush University Medical Center<br>Gray, Phillip; Ambry Genetics Corp<br>Cobleigh, Melody; Rush University Medical Center<br>Rao, Ruta; Rush University Medical Center<br>Patel, Jeremy; Rush University Medical Center<br>Kuzel, Timothy; Rush University Medical Center<br>Buckingham, Lela; Rush University Medical Center<br>Usha, Lydia; Rush University Medical Center
Keywords:	targeted therapy, precision medicine, breast cancer, clinical care, genetic testing
Abstract:	Background: Genetic testing for cancer predisposition is recommended to women with breast cancer who meet the criteria for such testing. After the FDA approvals of the Poly ADP Ribose Polymerase (PARP) inhibitors olaparib and talazoparib for treatment of metastatic breast cancer carrying germline mutations in BRCA1 and BRCA2 genes, the genetic testing result has become critical in their care. With the recent FDA approval of alpelisib for the treatment of PIK3CA-mutated hormone-receptor positive metastatic breast cancer, tumor molecular profiling to identify somatic mutations and potential molecularly targeted agents is increasingly utilized in the treatment of advanced breast cancer.<br>Aims: Combining germline and somatic sequencing (paired testing) offers an advantage over a single technique approach. Our study evaluates the role of paired testing on the management of breast cancer patients.<br>Methods and Results: 43 breast cancer patients treated at Rush University Medical Center underwent paired germline and somatic variant testing in 2015 -2017. A retrospective chart review was conducted with the analysis of demographic, clinical, and genomic data. Three actionable germline variants were found in the CHEK2 (2) and ATM (1) genes. 95% of tumors had somatic mutations. 77% of tumors had genomic

alterations targetable with agents approved for breast cancer and 88% had molecular targets for agents approved for other cancers. Clinical examples of such use are described and potential future directions of tumor and paired testing are discussed.
Conclusions: Germline variants were present in a relatively small patient group not routinely tested for inherited alterations. Potentially targetable somatic alterations were identified in the majority of breast cancers. Paired testing is a feasible and efficient approach that delivers valuable information for the care of breast cancer patients and eliminates serial testing.
Keywords: Breast cancer, genomic profiling, next-generation sequencing, germline testing, cancer predisposition, clinical utility

SCHOLARONE™
Manuscripts

Paired Tumor Sequencing and Germline Testing in Breast Cancer Management: An Experience of a Single Academic Center

Elizabeth Elliott[1], Virginia Speare[2], James Coggan[1], Carin Espenschied[2], Holly LaDuca[2], Amal F. Yussuf[2], Kelly Burgess[1], Phillip Gray[2], Melody Cobleigh[1], Ruta Rao[1], Jeremy Patel[1], Timothy Kuzel[1], Lela E. Buckingham[1], Lydia Usha[1]

[1]Rush University Medical Center, 1653 W Congress Pkwy, Chicago, IL 60612

[2]Ambry Genetics, Ambry Genetics, 15 Argonaut, Aliso Viejo, CA 92656, USA

For Review Only

Running Title: The use of combined tumor molecular profiling and germline testing in breast cancer

Financial Support: Rush Metastatic Breast Cancer Philanthropic Fund. Laboratory testing was performed by Ambry Genetics free of charge.

Corresponding Author: James Coggan, DO

1725 W. Harrison Street, Suite 809

Chicago, IL 60612

Ph: (312) 563-2200, fax: (312) 942-3192

Email: james_l_coggan@rush.edu

Abstract:

Background: Genetic testing for cancer predisposition is recommended to women with breast cancer who meet the criteria for such testing. After the FDA approvals of the Poly ADP Ribose Polymerase (PARP) inhibitors olaparib and talazoparib for treatment of metastatic breast cancer carrying germline mutations in *BRCA1* and *BRCA*2 genes, the genetic testing result has become critical in their care. With the recent FDA approval of alpelisib for the treatment of *PIK3CA*-mutated hormone-receptor positive metastatic breast cancer, tumor molecular profiling to

identify somatic mutations and potential molecularly targeted agents is increasingly utilized in the treatment of advanced breast cancer.

Aims: Combining germline and somatic sequencing (paired testing) offers an advantage over a single technique approach. Our study evaluates the role of paired testing on the management of breast cancer patients.

Methods and Results: 43 breast cancer patients treated at Rush University Medical Center underwent paired germline and somatic variant testing in 2015 -2017. A retrospective chart review was conducted with the analysis of demographic, clinical, and genomic data. Three actionable germline variants were found in the *CHEK2* (2) and *ATM* (1) genes. 95% of tumors had somatic mutations. 77% of tumors had genomic alterations targetable with agents approved for breast cancer and 88% had molecular targets for agents approved for other cancers. Clinical examples of such use are described and potential future directions of tumor and paired testing are discussed.

Conclusions: Germline variants were present in a relatively small patient group not routinely tested for inherited alterations. Potentially targetable somatic alterations were identified in the majority of breast cancers. Paired testing is a feasible and efficient approach that delivers valuable information for the care of breast cancer patients and eliminates serial testing.

Keywords: Breast cancer, genomic profiling, next-generation sequencing, germline testing, cancer predisposition, clinical utility

For Review Only

Introduction:

Recent advances in precision oncology allow for the administration of more effective and less toxic treatments, leading to improved patient outcomes. Molecularly targeted agents (MTAs) such as imatinib in chronic myelogenous leukemia or trastuzumab in Her-2/neu-positive breast cancer have shown significant prolongation of overall survival in the corresponding malignancies and sometimes, cures.[1] A meta-analysis of Phase I trials with more than 13,000 patients showed that biomarker-driven MTAs as compared to non-personalized treatments resulted in a higher response rates (30.6% vs 4.9%) and better progression-free survival (PFS; 5.7 mo vs 2.9 mo).[2] In addition, MTAs usually have fewer side effects (on-target or off-target) than cytotoxic agents. There is an ever-increasing number of clinical trials underway testing new targets and matching therapies.[3] In January of 2018, a PARP-inhibitor olaparib became the first genomically-based treatment approved by the United States FDA (Food and Drug Administration) for patients with metastatic breast cancer (MBC), specifically those who carry germline *BRCA1* or *BRCA2* mutations.[4,5] Subsequently, two more MTAs (talazoparib and alpelisib) have been FDA-approved for metastatic breast cancer with a germline *BRCA* mutation (talazoparib) and somatic *PIK3CA* alteration (alpelisib).[6,7] Advances such as these confirm the promise of molecularly based approaches in breast cancer (BC) management. Currently, therapy in BC is guided by the presence of estrogen and progesterone receptors as well as human epidermal growth factor receptor 2 (HER2) protein overexpression or amplification on pathologic examination.[8-11] The introduction of next-generation DNA sequencing (NGS) and recently, mapping , whole cancer genomes to find cancer "drivers", allowed to identify promising MTAs. [12, 13] At present, however, alpelisib is the only MTA which requires identification of *PIK3CA* mutation in the tumor to predict its efficacy. Tumor molecular profiling (TMP) is usually employed in advanced BC when all standard treatments have been exhausted.[14] Since the technology of TMP with NGS became available, many experts considered it investigational and appropriate only in a clinical trial setting. Yet in March of 2018 Centers for Medicare & Medicaid Services made the decision that NGS is "reasonable and necessary" in advanced cancer for Medicare beneficiaries, opening the doors for wider use of this laboratory testing.[15] NGS is also employed in germline hereditary predisposition testing, which typically involves the use of multi-gene panels on constitutional DNA isolated from blood cells. Germline testing provides valuable information on cancer surveillance and risk reduction for patients and their

For Review Only

biological relatives. It may also provide information for MTA selection, such as identifying a germline *BRCA1/2* mutation in a patient with metastatic BC who may benefit from treatment with Poly ADP Ribose Polymerase (PARP) inhibitors. Combining germline testing with TMP (paired testing or PT) may provide a time and resource efficient option to reveal molecular targets for therapy and genetic predisposition to cancer.[16]

We performed a pilot study of PT in a series of 43 BC patients to assess the feasibility and clinical impact of this testing approach at a single academic medical center. We discuss the implications of PT results for treatment selection, subsequent cancer surveillance, and screening of family members. We also discuss efficiency of the PT approach.

For Review Only

Methods:

We performed a retrospective chart review of women with the diagnosis of BC who underwent PT when deemed appropriate by their oncologists in the context of routine clinical care at Rush University Medical Center from November 2015 to February 2017. The following data were collected: patient age, ethnic background, clinical stage at diagnosis, histology, ER/PR/HER2 receptor status, BC treatment history, and PT results. All patients received pre-test risk assessment and genetic counseling to inform them about PT. Post-test genetic counseling was provided for patients and/or their families who tested positive for a pathogenic or likely pathogenic germline variant. The study was approved by the Rush University Institutional Review Board (IRB# 17041703-IRB01). Participants were deidentified before group statistical analyses.

Laboratory analysis

DNA was extracted from a primary tumor for the majority of subjects while a metastatic lesion was analyzed when the primary tumor was not available. Blood or saliva was also submitted for each patient for germline DNA extraction. Samples were processed as previously described.[16] PT consisted of a custom probe-based NGS tumor panel (Illumina HiSeq) for the detection of single nucleotide variants, small insertions and deletions in 142 genes that frequently harbor somatic and/or germline mutations in cancer (Online Resource Supplemental Table 1). The NGS panel used detects gene fusions and structural variants, such as tandem duplications and inversions, in 15 frequently disrupted oncogenes and tumor suppressors. Tumor tissue and a matched blood specimen were analyzed using a custom bioinformatics pipeline to differentiate between somatic and germline mutations, allowing for precise variant classification. Briefly, paired normal samples were analyzed using

Novoalign V3.02.07 to align FASTQ reads to a reference sequence (hg19) and GATK (V3.2.2) to generate variants and no/low coverage reports. Germline variants were filtered using a Q score of 30, coverage of 10X, het ratio of 10% and filtered out if determined to be a sequencing artifact or common polymorphism utilizing population frequency data from NCBI dbSNP, NHLBI Exome Sequencing Project (ESP), 1000 Genomes, and internal Ambry data. In tumor-normal analysis mode, Varscan2 (v2.3.6), was used to detect somatic variants as low as 3% minor allele frequency. Structural variants were annotated using Oncofuse v1.0.7 and DELLY v.0.6.1 respectively. Germline genetic variants were assessed using Ambry's five-tier classification framework based on guidelines published by the American College of Medical Genetics and Genomics and the Association for Molecular Pathology (pathogenic; likely pathogenic; variant of uncertain significance; likely benign; benign). [17,18] Tumor specimens were also analyzed using the Affymetrix OncoScan platform, an array technology for high resolution copy number variant detection that can detect single copy amplifications, hemizygous deletions and copy neutral loss of heterozygosity.

Alterations identified in TMP and associations with MTAs were reported based on peer reviewed studies and other publicly available resources. Germline variants when identified in blood or tumor were reported for the following genes: *APC, BRCA1, BRAC2, MLH1, MSH2, MSH6, MUTYH, PMS2, PTEN, RB1, RET, SDHAF2, SDHB, SDHC, SDHD, STK11, TP53, TSC1, TSC2,* and *VHL.* In some cases, germline testing was performed on additional cancer susceptibility genes as clinically indicated based on genetic risk assessment. Actionable germline alterations were defined as those alterations associated with a currently available option for cancer surveillance, prevention or treatment in either the patient or their close family members.

Results:

Age at diagnosis, ethnicity, stage at the time of testing, histologic type, and tumor markers of breast cancer for 43 patients are reported in Table 1. Our patient population had a greater proportion of stage IV disease at diagnosis and a younger age than average BC population when compared to National Cancer Institute (NCI) epidemiological data which may reflect the referral bias since patients with advanced disease were more likely to get referred for paired testing. [19,20] The primary histological BC subtype of this patient group was infiltrating ductal carcinoma (IDC), which correlates with NCI data, yet there was a somewhat larger proportion of infiltrating lobular

For Review Only

carcinoma, which may be the result of a small sample.[19] The majority of our patients had hormone receptor positive, HER2 negative BC, consistent with national data.[21]

Somatic genomic alterations in the tumors tested were highly prevalent with 95% (42/43) of tumors having at least one reported mutation with an average of 6.5 (range 0-17) mutations per tumor. Overall, 77% of tumors contained at least one target for BC therapy, and 88% contained an FDA-approved targeted therapy for another type of cancer, potentially available off-label for BC. (Table 2)

A total of 278 alterations in 81 genes were clinically reported in Figure 1, and are broken down by ER receptor status in Supplemental Graph 1. Hemizygous loss of a gene was the most common alteration (n=96) while missense alterations (n=79) and gene amplifications (n=66) were also frequent (Online Resource Supplemental Table 2). Genes known to be frequently mutated in BC (*BRCA1, BRCA2, CDH1*, *PIK3CA*, *PTEN*, and *TP53*) accounted for 40% of alterations. 22 genes altered in this cohort of patients had an associated MTA for breast cancer while alterations in additional genes added to the number of patients who were potentially eligible for an MTA (Figure 2). Patient characteristics in relation to the alterations can be found in Online Resource Supplemental Table 3.

Three patients were found to have actionable germline variants that were not previously suspected including one pathogenic mutation in *CHEK2* (c.1100delC), a likely pathogenic variant in the *CHEK2* gene (c.1427C>T), and a likely pathogenic splice site variant in the *ATM* gene (2466+1G>C). None of these three patients met the then current National Comprehensive Cancer Network (NCCN) germline genetic testing criteria.[22] We have performed TMP on breast cancers of the three germline carriers described above (Table 3). Two of these tumors had multiple molecular alterations.

*Clinical Application of PT Results***:**

Because 47% of our sample (20 patients) consisted of patients with early-stage breast cancer, real time clinical care was usually not impacted by PT but rather, adjuvant or neoadjuvant treatment was based on stage, pathologic characteristics of the tumor including tumor markers, and in some cases on the results of molecular prognostic assays such as Oncotype DX® and Mammaprint®. In one patient (Patient C), an MTA was considered in adjuvant setting and in two women (Patient A and Patient B), MTAs were administered when BC recurred.

For Review Only

Patient 404122 (Supplemental Table 3) was diagnosed with inflammatory triple-negative left-sided IDC, clinical stage III, grade 3, at age 66. She received four cycles of neoadjuvant dose-dense doxorubicin and cyclophosphamide, followed by four cycles of paclitaxel, and underwent left modified radical mastectomy which demonstrated significant residual BC: ypTis N3a. After radiation to the left chest wall, adjuvant chemotherapy with carboplatin was attempted but not tolerated due to rash and weakness. She was diagnosed with histologically confirmed recurrence of triple-negative BC and metastases to intra-thoracic lymph nodes a year after her surgery. PT was performed on the primary tumor and revealed four somatic genomic alterations including *KRAS* amplification, *BARD1* p.A724V, *TP53* p. K139_P142del, and *TP53* copy neutral LOH. Based on these findings, she was prescribed a suggested MTA off-label, Trametinib, a mitogen-activated protein kinase kinase (MEK) inhibitor which targets *KRAS* amplification. Although it does not directly inhibit MEK, Trametinib has been shown to limit tumor progression via CD8 T-cell mediated factors by altering signaling along the RAS-ERK pathway in cancers with *KRAS* amplification.[23] Unfortunately, trametinib was discontinued one month later due to progression.

Patient 435327 (Supplemental Table 3) was diagnosed with clinical stage III, grade 3, triple-negative right-sided IDC at age 62. She received four cycles of neoadjuvant dose-dense doxorubicin and cyclophosphamide, followed by 12 cycles of weekly paclitaxel. She underwent subsequent right modified radical mastectomy with residual tumor noted, ypT2N0. She then completed radiation and received six cycles of adjuvant chemotherapy with capecitabine. She also was diagnosed with BC recurrence and histologically-confirmed pulmonary metastases one year after surgery. She underwent PT using a tissue sample from her mastectomy, notable for four somatic genomic alterations: *PIK3R1* p.R461_E462delinsQ, *FGF3* amplification, *FGF4* amplification, and HER2-Neu (*ERBB2*) mutation, p.S310F. Given the finding of *HER2-Neu* mutation, she was offered participation in phase II study with neratinib. [24]. Because somatic *HER2-Neu* mutations activate the *HER2-Neu* oncogene without its amplification, Trastuzumab and other commonly used *HER2-Neu* directed agents are usually ineffective in *HER2-Neu*-mutated BC. Neratinib is a small molecule which irreversibly inhibits both HER2-Neu amplifications and mutations.[25] The patient enrolled in this trial but it was discontinued 3 months later due to cancer progression.

Patient 487820 (Supplemental Table 3) was diagnosed with clinical stage III, Grade 2, ER-positive, PR-negative, HER2-negative IDC with neuroendocrine features at age 52. She received neoadjuvant doxorubicin and cyclophosphamide, followed by dose-dense paclitaxel. She underwent total left mastectomy with sentinel lymph

node biopsy which showed residual BC, ypT1cN1. PT of her surgical sample revealed somatic mutations in *TP53* and a hemizygous loss of *NF2*, yielding four possible therapies including an mTOR inhibitor everolimus, approved in combination with anti-hormonal therapy for metastatic BC. Given the significant amount of residual disease and this molecular alteration, she was evaluated for the adjuvant trial with everolimus.[26] However, she was found to be ineligible because positive lymph node biopsy was not followed by axillary lymph node dissection based on patient and surgeon preference. She underwent adjuvant radiation therapy and started anti-hormonal therapy with tamoxifen. At the time of this writing, she remains without evidence of disease 3.5 years after surgery.

Discussion:

In our study, 95% of all tumors analyzed demonstrated at least one somatic mutation presumed to be involved in tumorigenesis. The most commonly detected mutation was in *TP53* gene, followed by *CDH1* and *PIK3CA*. This correlates with other studies, which have described *TP53* as the most commonly mutated gene in human cancers.[27] As seen in Figure 1, the rate of gene alteration in *TP53* was less than 12% and that in *PIK3CA* was less than 6%, which are lower than reported results for breast cancer. Those studies, however, include non-coding regions with high mutation frequencies not all of which can be classified as driver mutations. Mutational signatures extended to genome rearrangements, characterized by tandem duplications or deletions, appear to be associated with defective homologous recombination-based DNA repair.[28] Analysis of all classes of somatic mutation across exons, introns and intergenic regions might generate a higher overall mutation rate, not all of which will be driver mutations. In general, mutations in tumor suppressor genes (loss of function mutations) like *TP53* are more difficult to target than mutations in oncogenes (gain of function mutations).[29] PARP-inhibitors is the first class of MTA that targets mutations in tumor suppressor genes (*BRCA1* and *BRCA2*). Preliminary data suggest that these agents can also elicit synthetic lethality in the presence of mutations in other tumor suppressor such as *TP53* and *PTEN* which are mutated in 37% and 3% of all BCs respectively and represented 11.8% and 5.4% of mutations in our sample. [30,31]

Gain-of-function mutations in genes such as *PIK3CA* which allow unregulated cell proliferation had associated MTAs (mTOR inhibitors: temsirolimus and everolimus; recently approved *PIK3CA* inhibitor: alpelisib) while others, such as a loss-of-function mutations in the tumor suppressor gene *CDH1* were not targetable in our patients (Online Supplemental Table 2). Notably, only one of our tumor samples demonstrated a mutation of *ESR1*

For Review Only

(estrogen receptor 1). This may be explained by the number of primary, non-metastatic breast tumor tissue in our sample, as the incidence of *ESR1* mutations rises to 15-20% in metastatic ER positive tumors after prior endocrine treatments, suggesting the development of resistance. [32]

A molecular target for breast cancer treatment was available for a majority (33/43) of patients. However, we did not observe responses in two patients who received MTAs. This is consistent with initial results of clinical trials looking at the use of MTAs in prospective fashion.[33,34] For instance, in SAPHIR01 trial for women with metastatic BC, 13% of patients were able to receive MTAs based on the genomic data and of those patients, only 9% had objective tumor response.[35]

When analyzing the results of MTA clinical trials, it is important to remember that they depend on target identification in tumor tissue and availability of matching MTAs. In the initial phase of the NCI sponsored MATCH (Molecular Analysis for Therapy Choice) trial, only 9% of patients had a matched targeted therapy, when there were only 10 MTAs and 10 corresponding arms in the study. However, the percentage of patients increased to 23% when the number of MTAs and the respective arms in the study went up to 24% .[36]

Previous studies have revealed the prevalence of germline variants discovered in paired testing. A retrospective study of Stanford patients with *BRCA1/2* somatic mutations found 55.7% were positive for pathogenic *BRCA1/2* germline mutations, confirming "a second hit" hypothesis.[37] In a study of patients with advanced cancer diagnoses, presumed pathogenic germline variants were found in 17 of 269 (6.3%) breast cancer patients (6.3%).[38] In another study of 1040 breast, prostate, renal, pancreatic, and colon cancer patients, 182 (17.5%) had clinically actionable mutations conferring cancer susceptibility. Of these, 101 patients would not have had these mutations detected using clinical guidelines. Germline findings led to predictive testing in the families of 13 individuals (1.3%), including 6 for whom genetic evaluation would not have been initiated by guideline-based testing. [39] In our small sample, we identified three patients (7.0% of those tested) with previously unrecognized actionable germline mutations in *CHEK2* and *ATM* genes which is consistent with these data.

Variants in *CHEK2* and *ATM* can impact cancer surveillance and cancer screening and prevention for blood relatives.[40,41] For our patients, the discovery of these actionable germline mutations led to recommendations for increased breast cancer surveillance with the addition of screening breast MRIs (for both *ATM* and *CHEK2* mutation carriers) and more frequent colonoscopies for colon cancer surveillance (for *CHEK2* mutation carriers only). [42]

For Review Only

The same recommendations would apply to their blood relatives if they carry these mutations. Currently, germline genetic testing for hereditary predisposition to breast and other cancers is only offered to individuals who meet specific testing criteria set forth by different societal guidelines, most commonly, the National Comprehensive Cancer Network (NCCN) Clinical Practice Guidelines. The NCCN guidelines recommend genetic testing for *BRCA1/2* mutations based on age at breast cancer diagnosis and burden of breast, ovarian and other related cancers in the family. While these guidelines are useful to identify individuals who should be considered for genetic testing, germline mutations in the *BRCA1/2* and other BC susceptibility genes may be missed in individuals who do not meet these criteria as their sensitivity is limited.[43,44] PT may allow for detecting pathogenic germline mutations in such BC patients. Importantly, somatic *BRCA 1/2* mutations in the tumors of *BRCA* 1/2 mutation carriers can cause functional reversal of the germline mutation and restoration of the wild-type BRCA which results in resistance to Carboplatin and PARP-inhibitors in these patients. As opposed to “a second-hit” mutation which affects a normal allele, a “reversing” mutation usually affects the allele with a germline mutation.[45]

For Review Only

Ongoing studies evaluate a potential benefit of PARP-inhibitors in the presence of somatic *BRCA1/2* mutations as well as germline and somatic mutations in other genes in homologous recombination (HR) pathway, including *ATM* and *CHEK2*. In a Phase 2 Study of Olaparib Monotherapy in Metastatic Breast Cancer Patients With Germline or Somatic Mutations in DNA Repair Genes (Olaparib Expanded) the response to olaparib is studied in patients with metastatic BC and germline or somatic mutations in the following genes: *ATM, ATR, BARD1, BRIP1 (FANCJ), CHEK2 , FANCA, FANCC, FANCD2, FANCE, FANCF, FANCM, MRE11A, NBN, PALB2, RAD50, RAD51C, RAD51D*.[46] In our cohort of women, if their BC recurs, three germline mutation carriers can be candidates for this trial as well as seven patients with somatic mutations in at least one of the above listed genes.

The same genomic alterations can be a target for a chemotherapy agent carboplatin which is known to double the objective tumor response rate in women with metastatic BC and germline *BRCA1/2* mutations.[47] HR deficiency in early- stage BC as measured by a HRD (Homologous Recombination Deficiency) molecular assay was associated with a higher chance to benefit from Carboplatin in neoadjuvant setting.[48,49] In a study of triple negative breast cancer, 59% of cases with homologous-recombination-repair deficiency had better outcome on

adjuvant chemotherapy for invasive disease-free survival (hazard ratio 0.42) compared to those without, regardless of whether a genetic/epigenetic cause was identified. [13]

In our study, all three germline mutation carriers had mutations in HR pathway (*CHEK2 and ATM* genes). In addition, the somatic alterations in two of these three tumors also included many genes in HR pathway (*BRCA1 and BRCA2, NF1 and NF2, FANCA*) (Table 3). It is logical to suggest that these somatic alterations (frequently, hemizygous losses) in the same pathway played a key role in the tumor development initiated by the germline mutation. Given this constellation of germline and somatic variants, it is likely that these cancers would respond to PARP-inhibitors and carboplatin if they recur.

As discussed earlier, TMP and PT data are usually not incorporated in treatment decisions for early BC. However, this may change with the increased use of platinum agents for adjuvant and neoadjuvant treatment of BC in *BRCA1/2* mutation carriers. Also, clinical trials are looking at a potential benefit of PARP-inhibitors in adjuvant and neoadjuvant treatment of high-risk BC patients with these mutations.[50] A recent study showed that patients with BC harboring kinase or helical domain *PIK3CA* mutations derived significantly greater benefit from letrozole over tamoxifen in BIG 1-98 adjuvant trial.[51] If confirmed prospectively this molecular finding can inform the selection of adjuvant anti-hormonal agents in the future. An integrative analysis of 2,658 whole-cancer genomes and their matching normal tissues across 38 tumor types from the Pan-Cancer Analysis of Whole Genomes (PCAWG) Consortium found cancer genomes contained 4–5 driver mutations, however, in around 5% of cases no drivers were identified, suggesting that cancer driver discovery is not yet complete. [12]

Our study showed that the use of PT is feasible in an actual clinical setting and a single academic institution. Even though TMP can potentially identify germline mutations, their ascertainment as such is difficult without a paired germline testing. [52] PT is more efficient than a germline testing or tumor sequencing alone, providing valuable information for the care of BC patients and eliminating their "testing fatigue" when asked to do serial tests; however, larger prospective studies are needed to assess the clinical impact and cost-effectiveness of PT for women with newly-diagnosed BC.

<u>Conclusions</u>:

For Review Only

With the rising demand for targeted therapies and the rapidly-changing landscape of MTAs, it is obvious that tumor sequencing holds promise for BC. Outcomes of the three patients in this study treated with MTA based on somatic alterations demonstrate the limitations that remain for their current use. In the years to come, cancer treatment may be dictated more by tumor mutations than tumor type. Yet, before BC tumor sequencing and paired testing become routine, active targets must be validated and more MTAs developed, including those that aim at targets currently deemed "not-druggable." Achieving these goals is likely to improve insurance reimbursement for BC sequencing and PT which at present, is often lacking.

Clinical trials are desperately needed to evaluate the efficacy and safety of MTAs. However, enrollment into these trials can be challenging as they are increasingly conducted in smaller biomarker-enriched patient populations selected by the presence of the target. In addition, since <1% - 20% of tumors harbor individually rare somatic mutations", collecting and reporting individual responses to MTAs as described in this paper ("N-of-one experiences") are crucial for future success of MTAs.[53] For this reason, we support the establishment of an up-to-date Web-based open-access database of molecular targets and responses to MTAs in individual patients such as the ASCO initiative CancerLinQ. [54]

Analysis of cancer-related genes in paired germline and tumor DNA samples can lead to increased detection of clinically significant heritable mutations compared to the predicted yield of targeted germline testing based on current clinical guidelines. Identification of germline variants can help guide therapeutic and preventive interventions. Drawing from our experience in this study, we predict expanding indications for paired somatic and germline testing in the future for BC as well as other types of cancer.

For Review Only

Ethical Statement

The study was approved by the Rush University Institutional Review Board (IRB# 17041703-IRB01).

Data Availability Statement

Our data is not currently shared in a repository, but can be made available upon request.

Acknowledgement

We would like to acknowledge the generous contributions of Mr. and Mrs. Eugene Deutsch, Mr. and Mrs. Mark Ditsch, Mr. George Ruwe, Mr. and Mrs. Douglas Criner, which made this work possible.

Conflicts of Interest

At the time research was conducted, Virginia Speare, Holly LaDuca, Carin Espenschied, Amal Yussuf, and Philip Gray were full time employees of Ambry Genetics. Carin Espenschied is a current employee and stock holder at Guardant Health. Otherwise we have no other conflicts of interest to report.

Authors' Contributions

All authors had full access to the data in the study and take responsibility for the integrity of the data and the accuracy of the data analysis. *Conceptualization*, L.U., C.E., PG; *Methodology*, E.E., V.S, K.B., P.G., C.E., L.U.; *Investigation*, E.E., M.C., R.R., K.B., H.L., A.Y., L.U.; *Formal Analysis*, P.G., A.Y., V.S.; *Resources*, M.C., R.R., P.G., L.U; *Writing – Original Draft*, E.E., J.C., J.P., K.B., and L.U.; *Writing – Review & Editing*, E.E., J.C., J.P., K.B., V.S., C.E., H.L., A.Y., P.G., T.K., L.B., H.L., M.C., R.R, and L.U.; *Visualization*, C.E., A.Y., V.S, J.C.; *Supervision*, C.E., L.U., V.S.; *Funding Acquisition*, L.U., H.L., P.G., V.S.

For Review Only

For Review Only

References:

For Review Only

1. Hochhaus A., Larson R.A., Guilhot F., Radich J.P., Branford S., Hughes T.P., Baccarani M., Deininger M.W., Cervantes F., Fujihara S., Ortmann C.-E., Menssen H.D., Kantarjian H., O'Brien S.G., Druker B.J., for the I.I. (2017) Long-term outcomes of imatinib treatment for chronic myeloid leukemia. The New England Journal of Medicine 376 (10):917-927. doi:10.1056/NEJMoa1609324
2. Schwaederle M., Zhao M., Lee J.J., Eggermont A.M., Schilsky R.L., Mendelsohn J., Lazar V., Kurzrock R. (2015) Impact of precision medicine in diverse cancers: A meta-analysis of phase ii clinical trials. Journal of Clinical Oncology 33 (32):3817-3825. doi:10.1200/JCO.2015.61.5997
3. Tsimberidou A.-M. (2015) Targeted therapy in cancer. Cancer Chemotherapy and Pharmacology 76 (6):1113-1132. doi:10.1007/s00280-015-2861-1
4. Robson M., Im S.-A., Senkus E., Xu B., Domchek S.M., Masuda N., Delaloge S., Li W., Tung N., Armstrong A., Wu W., Goessl C., Runswick S., Conte P. (2017) Olaparib for metastatic breast cancer in patients with a germline brca mutation. New England Journal of Medicine 377 (6):523-533. doi:10.1056/NEJMoa1706450
5. Administration U.S.F.D. (2018) Fda approves olaparib for germline brca-mutated metastatic breast cancer. https://www.fda.gov/drugs/informationondrugs/approveddrugs/ucm592357.htm. Accessed April 16, 2018
6. Talazaparib [package insert]. New York, NY. Pfizer. 2018.
7. Apelisib [package insert]. East Hannover, NJ. Novartis. 2019. 8. Rakha E.A., Starczynski J., Lee A.H.S., Ellis I.O. (2014) The updated asco/cap guideline recommendations for her2 testing in the management of invasive breast cancer: A critical review of their implications for routine practice. Histopathology 64 (5):609-615. doi:doi:10.1111/his.12357
9. Hammond M.E.H., Hayes D.F., Dowsett M., Allred D.C., Hagerty K.L., Badve S., Fitzgibbons P.L., Francis G., Goldstein N.S., Hayes M., Hicks D.G., Lester S., Love R., Mangu P.B., McShane L., Miller K., Osborne C.K., Paik S., Perlmutter J., Rhodes A., Sasano H., Schwartz J.N., Sweep F.C.G., Taube S., Torlakovic E.E. et al (2010) American society of clinical oncology/college of american pathologists guideline recommendations for immunohistochemical testing of estrogen and progesterone receptors in breast cancer. Journal of Clinical Oncology 28 (16):2784-2795. doi:10.1200/JCO.2009.25.6529
10. Krishnamurti U., Silverman J.F. (2014) Her2 in breast cancer: A review and update. Advances in Anatomic Pathology 21 (2):100-107. doi:10.1097/PAP.0000000000000015
11. Desch C.E., McNiff K.K., Schneider E.C., Schrag D., McClure J., Lepisto E., Donaldson M.S., Kahn K.L., Weeks J.C., Ko C.Y., Stewart A.K., Edge S.B. (2008) American society of clinical oncology/national comprehensive cancer network quality measures. Journal of Clinical Oncology 26 (21):3631-3637. doi:10.1200/jco.2008.16.5068
12**.** Campbell, P.J., Getz, G., Korbel, J.O. *et al.* Pan-cancer analysis of whole genomes. *Nature* **578,** 82–93 (2020). https://doi.org/10.1038/s41586-020-1969-6
13**.** Staaf, J., Glodzik, D., Bosch, A. *et al.* Whole-genome sequencing of triple-negative breast cancers in a population-based clinical study. *Nat Med* **25,** 1526–1533 (2019). https://doi.org/10.1038/s41591-019-0582-4
14. Ian S. Hagemann (2016) Molecular testing in breast cancer: A guide to current practices. Archives of Pathology and Laboratory Medicine 140 (8):815-824. doi:10.5858/arpa.2016-0051-RA
15. Center for Medicare and Medicaid Services (2018) Decision memo for next generation sequencing (ngs) for medicare beneficiaries with advanced cancer (cag-00450n). https://www.cms.gov/medicare-coverage-database/details/nca-decision-memo.aspx?NCAId=290. Accessed July 1, 2018
16. Gray P.N., Vuong H., Tsai P., Lu H.M., Mu W., Hsuan V., Hoo J., Shah S., Uyeda L., Fox S., Patel H., Janicek M., Brown S., Dobrea L., Wagman L., Plimack E., Mehra R., Golemis E.A., Bilusic M., Zibelman M., Elliott A. (2016) Tumornext: A comprehensive tumor profiling assay that incorporates high resolution copy number analysis and germline status to improve testing accuracy. Oncotarget 7 (42):68206-68228. doi:10.18632/oncotarget.11910
17. Pesaran T, Karam R, Huether R, et al. Beyond DNA: An Integrated and Functional Approach for Classifying Germline Variants in Breast Cancer Genes. *Int J Breast Cancer*. 2016;2016:2469523. doi:10.1155/2016/2469523
18. Richards S, Aziz N, Bale S, et al. Standards and guidelines for the interpretation of sequence variants: a joint consensus recommendation of the American College of Medical Genetics and Genomics and the Association for Molecular Pathology. *Genet Med*. 2015;17(5):405-424
19. National Cancer Institute Surveillance Epidemiology and End Results (SEER) Program (Released April 2017, based on the November 2016 submission). https://www.seer.cancer.gov. Accessed October 24 2017

For Review Only

20. Iqbal J., Ginsburg O., Rochon P.A., Sun P., Narod S.A. (2015) Differences in breast cancer stage at diagnosis and cancer-specific survival by race and ethnicity in the united states. JAMA 313 (2):165-173. doi:10.1001/jama.2014.17322
21. Howlader N., Altekruse S.F., Li C.I., Chen V.W., Clarke C.A., Ries L.A.G., Cronin K.A. (2014) Us incidence of breast cancer subtypes defined by joint hormone receptor and her2 status. JNCI Journal of the National Cancer Institute 106 (5):dju055. doi:10.1093/jnci/dju055
22. NCCN Referenced with permission from the NCCN Clinical Practice Guidelies in Oncology (NCCN Guidelines®) for genetic/familial high-risk assessment: Breast and Ovarian v.2.2019. © National Comprehensive Cancer network, Inc. 2018. All rights reserved. Accessed July 30, 2019. To view the most recent and complete version of the guideline, go online to NCCN.org. NCCN makes no warranties of any kind whatsoever regarding their content, use or application and disclaims any responsibility for their application or use in any way. https://www.nccn.org/professionals/physician_gls/pdf/genetics_screening.pdf.
23. Allegrezza M.J., Rutkowski M.R., Stephen T.L., Svoronos N., Perales-Puchalt A., Nguyen J.M., Payne K.K., Singhal S., Eruslanov E.B., Tchou J., Conejo-Garcia J.R. (2016) Trametinib drives t cell-dependent control of k-ras-mutated tumors by inhibiting pathological myelopoiesis. Cancer Research 76 (21):6253-6265. doi:10.1158/0008-5472.CAN-16-1308
24. Ma C.X., Bose R., Gao F., Freedman R.A., Pegram M.D., Blackwell K., Bedard P.L., Hayes D.F., Goetz M.P., Niravath P.A., Russell C.A., Tripathy D., Cobleigh M.A., Forero A., Pluard T.J., Anders C.K., Bumb C., Naughton M., Al-Kateb H., Ellis M.J. (2016) Phase ii trial of neratinib for her2 mutated, non-amplified metastatic breast cancer (her2mut mbc). Journal of Clinical Oncology 34 (15_suppl):516-516. doi:10.1200/JCO.2016.34.15_suppl.516
25. Bose R., Kavuri S.M., Searleman A.C., Shen W., Shen D., Koboldt D.C., Monsey J., Goel N., Aronson A.B., Li S., Ma C.X., Ding L., Mardis E.R., Ellis M.J. (2013) Activating her2 mutations in her2 gene amplification negative breast cancer. Cancer Discovery 3 (2):224-237. doi:10.1158/2159-8290.CD-12-0349
26. S1207 hormone therapy with or without everolimus in treating patients with breast cancer (2018) National Library of Medicine. https://ClinicalTrials.gov/show/NCT01674140.
27. Duffy M.J., Synnott N.C., Crown J. (2017) Mutant p53 as a target for cancer treatment. European Journal of Cancer 83:258-265. doi:10.1016/j.ejca.2017.06.023
28. Nik-Zainal S., Davies H., Staaf J., et. al. (2016). Landscape of somatic mutations in 560 breast cancer whole-genome sequences. Nature 534(7605):47-54. doi: 10.1038/nature17676. Epub 2016 May 2.
29. Guo X.E., Ngo B., Modrek A.S., Lee W.H. (2014) Targeting tumor suppressor networks for cancer therapeutics. Current Drug Targets 15 (1):2-16
30. Mendes-Pereira A.M., Martin S.A., Brough R., McCarthy A., Taylor J.R., Kim J.-S., Waldman T., Lord C.J., Ashworth A. (2009) Synthetic lethal targeting of pten mutant cells with parp inhibitors. EMBO Molecular Medicine 1 (6-7):315-322. doi:10.1002/emmm.200900041
31. The Cancer Genome Atlas N., Koboldt D.C., Fulton R.S., McLellan M.D., Schmidt H., Kalicki-Veizer J., McMichael J.F., Fulton L.L., Dooling D.J., Ding L., Mardis E.R., Wilson R.K., Ally A., Balasundaram M., Butterfield Y.S.N., Carlsen R., Carter C., Chu A., Chuah E., Chun H.-J.E., Coope R.J.N., Dhalla N., Guin R., Hirst C., Hirst M. et al (2012) Comprehensive molecular portraits of human breast tumours. Nature 490:61. doi:10.1038/nature11412
https://www.nature.com/articles/nature11412#supplementary-information
32. Jeselsohn R., De Angelis C., Brown M., Schiff R. (2017) The evolving role of the estrogen receptor mutations in endocrine therapy-resistant breast cancer. Current Oncology Reports 19 (5):35. doi:10.1007/s11912-017-0591-8
33. Astsaturov I. (2017) Future clinical trials: Genetically driven trials. Surgical Oncology Clinics of North America 26 (4):791-797. doi:10.1016/j.soc.2017.05.014
34. Le Tourneau C, Delord J-P, Goncalves A, et al. Molecular Targeted Therapy Based on Tumour Molecular Profiling Versus Conventional Chemotherapy for Advanced Cancer (SHIVA): A Multicentre, Open-Label, Proof-of-Concept, Randomised, Controlled, Phase 2 Trial. *Lancet Oncol*. 2015;16(13):1324-1334. doi:10.1016/S1470-2045(15)00188-6
35. Andre F., Bachelot T., Commo F., Campone M., Arnedos M., Dieras V., Lacroix-Triki M., Lacroix L., Cohen P., Gentien D., Adelaide J., Dalenc F., Goncalves A., Levy C., Ferrero J.M., Bonneterre J., Lefeuvre C., Jimenez M., Filleron T., Bonnefoi H. (2014) Comparative genomic hybridisation array and DNA sequencing to

For Review Only

direct treatment of metastatic breast cancer: A multicentre, prospective trial (safir01/unicancer). Lancet Oncology 15 (3):267-274. doi:10.1016/S1470-2045(13)70611-9

36. ECOG-ACRIN (2016) Executive summary: Interim analysis of the nci-match trial. Available via ECOG-ACRIN Cancer Research Group. http://ecog-acrin.org/nci-match-eay131/interim-analysis. Accessed August 18 2018

37. Vlessis K, Purington N, Chun N, et al.Germline Testing for Patients With *BRCA1/2* Mutations on Somatic Tumor Testing, *JNCI Cancer Spectrum*, Volume 4, Issue 1, February 2020, pkz095

38. Schrader K.A., Cheng D.T., Joseph V., et. al. (2016). Germline variants in targeted tumor sequencing using matched normal DNA. JAMA Oncology 2(1):104-11. doi: 10.1001/jamaoncol.2015.5208

39. Mankelker D., Zhang L., Kemel Y., et. al. (2017). Mutation detection in patients with advanced cancer by universal sequencing of cancer-related genes in tumor and normal DNA vs. guideline-based germline testing. JAMA 318(9):825-835. doi: 10.1001/jama.2017.11137.

40. Yadav S., Reeves A., Campian S., Sufka A., Zakalik D. (2017) Preoperative genetic testing impacts surgical decision making in brca mutation carriers with breast cancer: A retrospective cohort analysis. Hereditary Cancer in Clinical Practice 15:11. doi:10.1186/s13053-017-0071-z

41. Jerzak K.J., Mancuso T., Eisen A. (2018) Ataxia–telangiectasia gene (atm) mutation heterozygosity in breast cancer: A narrative review. Current Oncology 25 (2):e176-e180. doi:10.3747/co.25.3707

42. NCCN (2018) Referenced with permission from the NCCN Clinical Practice Guidelines in Oncology (NCCN Guidelines®) for Genetic/Familial High-Risk Assessment: Colorectal v.1.2018 © National Comprehensive Cancer Network, Inc 2018. All rights reserved. Accessed July 12, 2018. To view the most recent and complete version of the guideline, go online to NCCN.org. NCCN makes no warranties of any kind whatsoever regarding their content, use or application and disclaims any responsibility for their application or use in any way. https://www.nccn.org/professionals/physician_gls/pdf/genetics_colon.pdf. Accessed July 12 2018

43. Xie Y., Li G., Chen M., Guo X., Tang L., Luo X., Wang S., Yi W., Dai L., Wang J. (2018) Mutation screening of 10 cancer susceptibility genes in unselected breast cancer patients. Clinical Genetics 93 (1):41-51. doi:doi:10.1111/cge.13063

44. Koumpis C., Dimitrakakis C., Antsaklis A., Royer R., Zhang S., Narod S.A., Kotsopoulos J. (2011) Prevalence of brca1 and brca2 mutations in unselected breast cancer patients from greece. Hereditary Cancer in Clinical Practice 9 (1):10-10. doi:10.1186/1897-4287-9-10

45. Norquist B, Wurz KA, Pennil CC, et al. Secondary somatic mutations restoring BRCA1/2 predict chemotherapy resistance in hereditary ovarian carcinomas. *J Clin Oncol*. 2011;29(22):3008-3015. doi:10.1200/JCO.2010.34.2980

46. Olaparib in breast cancer (2018) National Library of Medicine. https://clinicaltrials.gov/ct2/show/NCT03344965. Accessed September 5, 2018

47. Tutt A., Tovey H., Cheang M.C.U., Kernaghan S., Kilburn L., Gazinska P., Owen J., Abraham J., Barrett S., Barrett-Lee P., Brown R., Chan S., Dowsett M., Flanagan J.M., Fox L., Grigoriadis A., Gutin A., Harper-Wynne C., Hatton M.Q., Hoadley K.A., Parikh J., Parker P., Perou C.M., Roylance R., Shah V. et al (2018) Carboplatin in brca1/2-mutated and triple-negative breast cancer brcaness subgroups: The tnt trial. Nature Medicine 24 (5):628-637. doi:10.1038/s41591-018-0009-7

48. Minckwitz G.V., Timms K., Untch M., Elkin E.P., Fasching P.A., Schneeweiss A., Salat C., Rezai M., Blohmer J.U., Zahm D.M., Jackisch C., Gerber B., Klare P., Kümmel S., Eidtmann H., Paepke S., Reid J.E., Nekljudova V., Hartman A.-R., Loibl S. (2015) Prediction of pathological complete response (pcr) by homologous recombination deficiency (hrd) after carboplatin-containing neoadjuvant chemotherapy in patients with tnbc: Results from geparsixto. Journal of Clinical Oncology 33 (15_suppl):1004-1004. doi:10.1200/jco.2015.33.15_suppl.1004

49. Telli M.L., Timms K.M., Reid J., Hennessy B., Mills G.B., Jensen K.C., Szallasi Z., Barry W.T., Winer E.P., Tung N.M., Isakoff S.J., Ryan P.D., Greene-Colozzi A., Gutin A., Sangale Z., Iliev D., Neff C., Abkevich V., Jones J.T., Lanchbury J.S., Hartman A.-R., Garber J.E., Ford J.M., Silver D.P., Richardson A.L. (2016) Homologous recombination deficiency (hrd) score predicts response to platinum-containing neoadjuvant chemotherapy in patients with triple-negative breast cancer. Clinical Cancer Research 22 (15):3764-3773. doi:10.1158/1078-0432.ccr-15-2477

50. Litton J.K., Scoggins M., Ramirez D.L., Murthy R.K., Whitman G.J., Hess K.R., Adrada B.E., Moulder S.L., Barcenas C.H., Valero V., Gomez J.S., Mittendorf E.A., Thompson A., Helgason T., Mills G.B., Piwnica-Worms H., Arun B.K. (2017) A feasibility study of neoadjuvant talazoparib for operable breast cancer patients with a germline brca mutation demonstrates marked activity. npj Breast Cancer 3 (1):49. doi:10.1038/s41523-017-0052-4

For Review Only

51. Luen S.J., Asher R., Lee C., et al. (2018) Association of somatic driver alterations with prognosis in postmenopausal, hormone receptor–positive, her2-negative early breast cancer: A secondary analysis of the big 1-98 randomized clinical trial. JAMA Oncology. doi:10.1001/jamaoncol.2018.1778
52. Ngeow, J., Eng, C. Precision medicine in heritable cancer: when somatic tumour testing and germline mutations meet. *npj Genomic Med* **1,** 15006 (2016). https://doi.org/10.1038/npjgenmed.2015.6
53. Santarpia L., Bottai G., Kelly C.M., Győrffy B., Székely B., Pusztai L. (2016) Deciphering and targeting oncogenic mutations and pathways in breast cancer. The Oncologist 21 (9):1063-1078. doi:10.1634/theoncologist.2015-0369
54. Oncology A.S.o.C. (2018) Asco cancerlinq. Available via American Society of Clinical Oncology. https://cancerlinq.org/. Accessed July 1 2018

For Review Only

Tables and Figures:

Table 1: Demographic and Clinical Characteristics of Breast Cancer Cases		
**Patient Characteristics**		**Patients with MTA for Breast Cancer (%)**
**Number of patients**	43	33/43 (76.7)
**Median Age at Diagnosis (IQR[a]) [years]**	66 (53, 72)	-
**Median Age at Tumor Testing (IQR) [years]**	64 (51, 70.5)	-
**Age at Diagnosis Group**	N (%)	
30-39	1 (2.3)	1/1 (100.0)
40-49	7 (16.3)	2/7 (28.6)
50-59	10 (23.3)	8/10 (80.0)
60-69	12 (27.9)	9/12 (75.0)
70-79	6 (14.0)	6/6 (100.0)
80 and over	7 (16.3)	6/7 (85.7)
**Race/Ethnicity (%)**		
Caucasian	23 (53.4)	19/23 (82.6)
Ashkenazi Jewish	0 (0.0)	-
African American	12 (27.9)	9/12 (75.0)
Asian	1 (2.3)	0/1 (0)
Hispanic	4 (9.3)	4/4 (100.0)
Multiple/Other/Unknown	3 (7.0)	1/3 (33.3)
**Clinical Stage at Time of Paired Testing (%)**		
I	4 (9.3)	3/4 (75)
II	6 (14.0)	5/6 (83.3)

For Review Only

III	4 (9.3)	2/4 (50.0)
IV	23 (53.4)	20/23 (87.0)
Incomplete early stage [b]	6 (14.0)	3/6 (50.0)
**Histology**		
IDC	29 (67.4)	26/29 (90.0)
ILC	7 (16.3)	5/7 (71.4)
Mixed Histology	6 (14.0)	1/6 (16.7)
Unknown Histology	1 (2.3)	1/1 (100.0)
**Receptor Status**		
Triple Negative (ER-/PR-/Her2-)	5 (11.6)	2/5 (40.0)
(ER+/PR+/Her2+) or (ER+/PR-/Her2+) or (ER-/PR+/Her2+)	6 (14.0)	3/6 (50.0)
(ER+/PR+/Her2-) or (ER+/PR-/Her-2-) or (ER-/PR+/Her2-)	28 (65.1)	26/28 (93.0)
(ER-/PR-/Her2+)	4 (9.3)	4/4 (100.0)
[a] IQR=interquartile range<br>[b] T1 - T2 tumors without nodal assessment		

Table 1 depicts demographic and clinical characteristics of the breast cancer cases included in this study.

For Review Only

Table 2: Genomic Alterations and Available Therapy and Clinical Trials	
**Genomic Alterations (Patients)[a]**	278 (43)
Patients with at least one FDA approved therapy for breast cancer (% of tumors affected)	33 (76.7)
Patients with at least one FDA approved therapy for other tumor type (% of tumors affected)	38 (88.3)
Patients who were eligible for a clinical trial based on genomic alteration(s) (% of tumors affected)	40 (93.2)

Table 2 depicts clinical implications of genomic alterations identified. It stratifies results based on percentage of total alterations meeting specific clinical criteria, and percentage of tumors included in the study which meet clinical criteria mentioned

[a] Gene alterations found in multiple patients were counted separately.

For Review Only

Table 3: Somatic Variants in Tumors of 3 Identified Germline Carriers

Patient	Germline Mutation	Somatic Variants	Selected Demographic and Clinical Characteristics. Disease Status as of July 2020
1	*CHEK2*, likely pathogenic c.1427C>T (p.Thr476Met)	4 somatic single nucleotide variants (SNVS):<br>*PIK3CA* p. H1047L<br>*AR* p.R13W<br>*TP53*, p. Y220C<br>*CDH1* p.N297S	Caucasian<br>Diagnosed in 2011 at age 64 with stage I breast cancer ER and PR positive, Her-2/neu negative.<br>Currently, NED<br>Also, has a history of clear cell papillary renal cell carcinoma
2	*ATM*, likely pathogenic c.2466+1G>C (splicing site exon 18)	10 somatic alterations:<br>*CDKN2A* hemizygous loss<br>*CDKN2B* hemizygous loss<br>*PTEN* hemizygous loss<br>*NF2* hemizygous loss<br>*BRCA2* hemizygous loss<br>*ATM* LOH<br>*FANCA* hemizygous loss<br>*FLCN* hemizygous loss<br>*T53* (pT155_R156del and T53 LOH)	Caucasian<br>Diagnosed in 2015 at age 50 with stage II breast cancer, ER positive, PR and Her-2/neu negative.<br>Currently, NED.
3	*CHEK2*, pathogenic c.1100del (pT367Mfs*15)	13 somatic alterations:<br>*CDKN2A* hemizygous loss<br>*CDKN2B* hemizygous loss<br>*PTEN* hemizygous loss<br>*NF1* hemizygous loss<br>*BRCA1* hemizygous loss<br>*ATM* hemizygous loss<br>*FLCN* hemizygous loss<br>*T53* hemizygous loss<br>*CDH1* hemizygous loss<br>*TSC1* hemizygous loss<br>*APC* hemizygous loss<br>*CDK4* amplification<br>*FRS2* amplification	African-American<br>Diagnosed in 2016 at age 79 with stage II breast cancer, ER positive, PR and Her-2/neu negative<br>Died in 2019 with no evidence of breast cancer recurrence.

Table 3 captures somatic variants and selected clinical and demographic information for the three patients with germline variants that were not previously suspected.

For Review Only

Figure 1: Frequency of Somatic Alterations by Gene

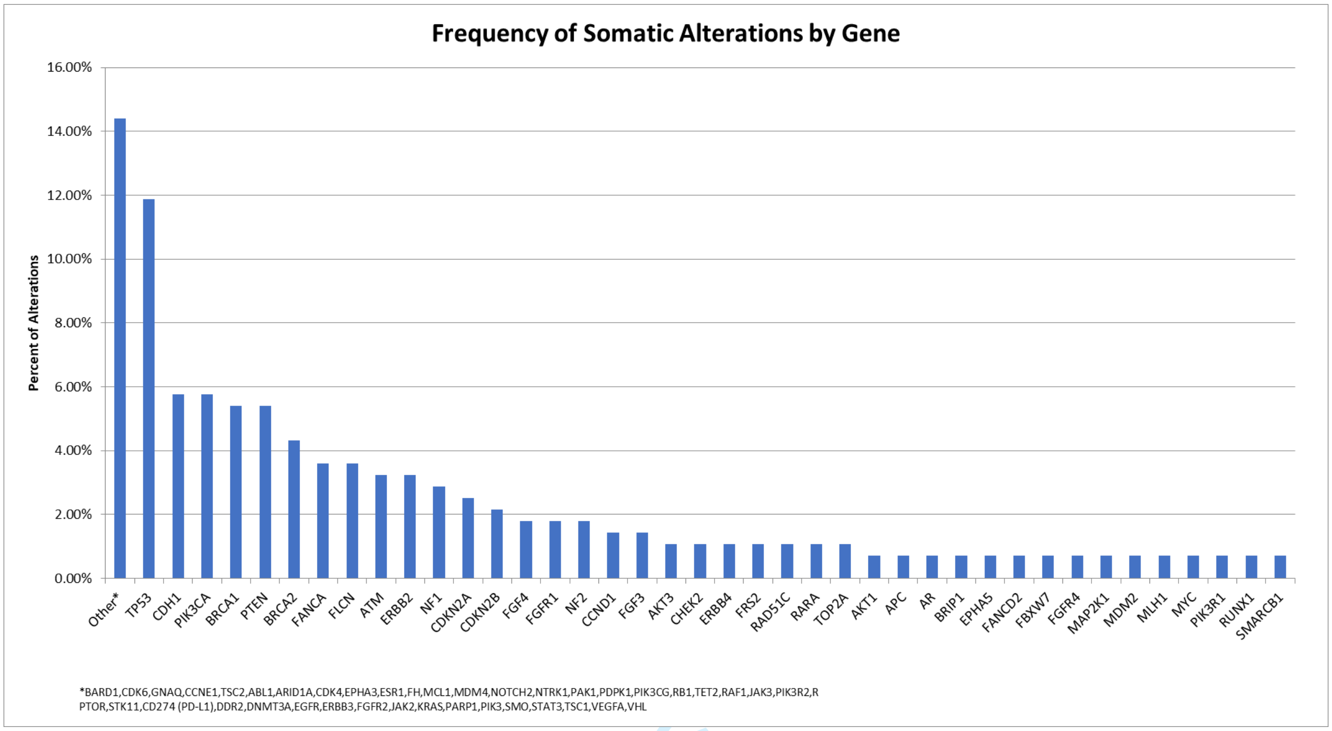

Figure 1 depicts frequency of specific genomic mutations identified across all tumors studied. 278 somatic alterations (mutations, allelic loss and amplifications) in 81 genes were clinically reported. The single most frequently observed gene was *TP53*. Genes known to be frequently mutated in BC (*BRCA1, BRCA2, CDH1*, *PIK3CA*, *PTEN*, including *TP53*) accounted for 40% of alterations.

w Only

Figure 2: Genes with Associated FDA Approved Therapy

A.

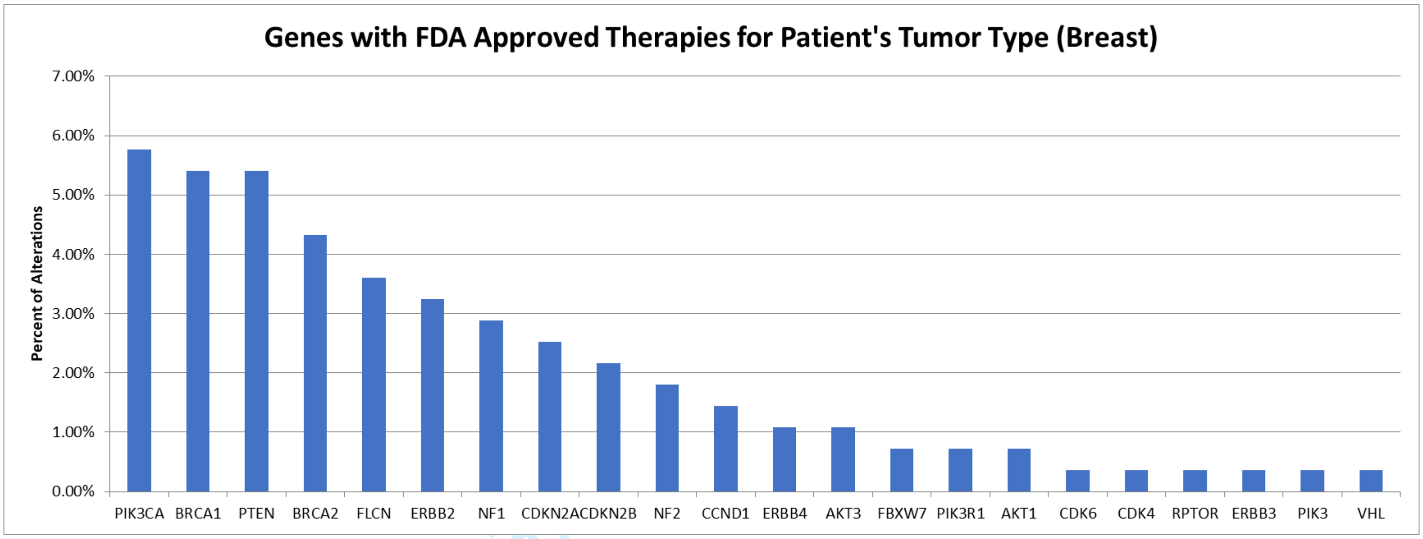

B.

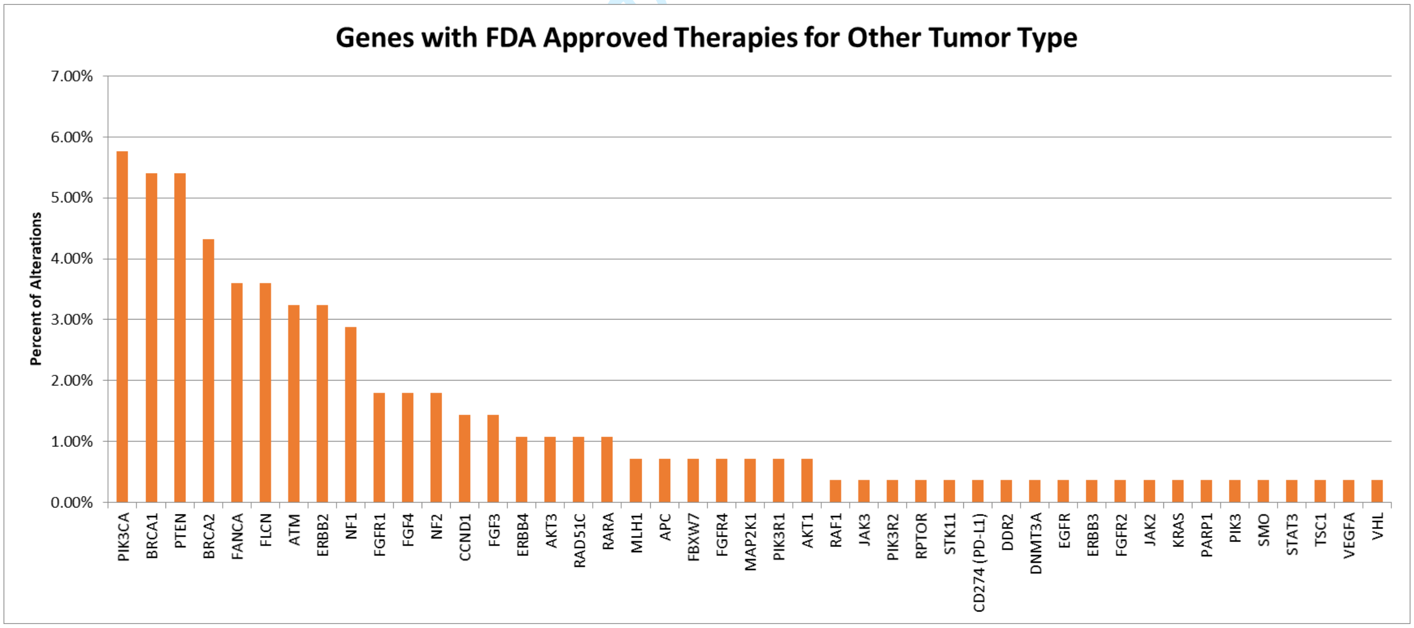

Figure 2 depicts frequency of genomic mutations with FDA approved therapies (both for breast cancer and for other tumor types) identified across all tumors studied. 22 genes altered in this patient group had an associated MTA for breast cancer (A). Alterations in additional genes had an associated MTA for other cancers (B).

Supplemental Table 1. TumorNext Solid Tumor Panel

Genes with full exon coverage							
ABL1	BCL2L1	CSF1R	FANCA	GNAQ	MAP2K2	MYD88	PDGFRB
ABL2*	BCL2L2	CTNNB1	FANCC	GNAS	MAP2K4	NBN	PIK3CA
AKT1	BMPR1A	DDR2	FANCD2	HRAS	MAX	NF1	PIK3CB
AKT2	BRAF	DNMT3A	FBXW7	HSP90AA1	MDM2	NF2	PIK3CG
AKT3	BRCA1	EGFR	FGFR1	IDH1	MET	NOTCH1	PIK3R1
ALK	BRCA2	EPHA2	FGFR2	IDH2	MITF	NOTCH2	PIK3R2**
APC	BRIP1	EPHA3	FGFR3	IGF1R	MLH1	NOTCH3	PMS2***
AR	BTK	EPHA5	FGFR4	IL7R	MLL	NOTCH4	PTCH1
ARAF	CBL	EPHA7	FH	JAK1	MPL	NRAS	PTEN
ATM	CCND1	EPHB1	FLCN	JAK2	MRE11A	NTRK1	PTPN11
AURKA	CCND3	ERBB2	FLT1	JAK3	MSH2	PAK7	RAD50
AURKB	CDH1	ERBB3	FLT3	KDR	MSH6	PALB2	RAD51C
AXL	CDK4	ERBB4	FLT4	KIT	MTOR	PARP1	RAD51D
BARD1	CDKN2A	ESR1	FOXL2	KRAS	MUTYH	PARP2	RAF1
BCL2	CHEK2	EZH2	GNA11	MAP2K1	MYCN	PDGFRA	RARA
Genes analyzed for structural variants							
ALK	BRAF	BRCA2	FGFR2	KIT	NTRK1	RAF1	ROS1
BCL2	BRCA1	FGFR1	FGFR3	NOTCH2	PDGFRA	RET	

*ABL2 exon 2 is not included in the assay

**PIK3R2 exon 13 is not included in the assay

***Only *PMS2* exons 1-10 are analyzed

For Review Only

RB1	TET2
RET	TMEM127
ROS1	TOP1
RUNX1	TP53
SDHA	TSC1
SDHAF2	TSC2
SDHB	VHL
SDHC	
SDHD	
SMAD4	
SMARCB1	
SMO	
SRC	
STAT3	
STK11	

For Review Only

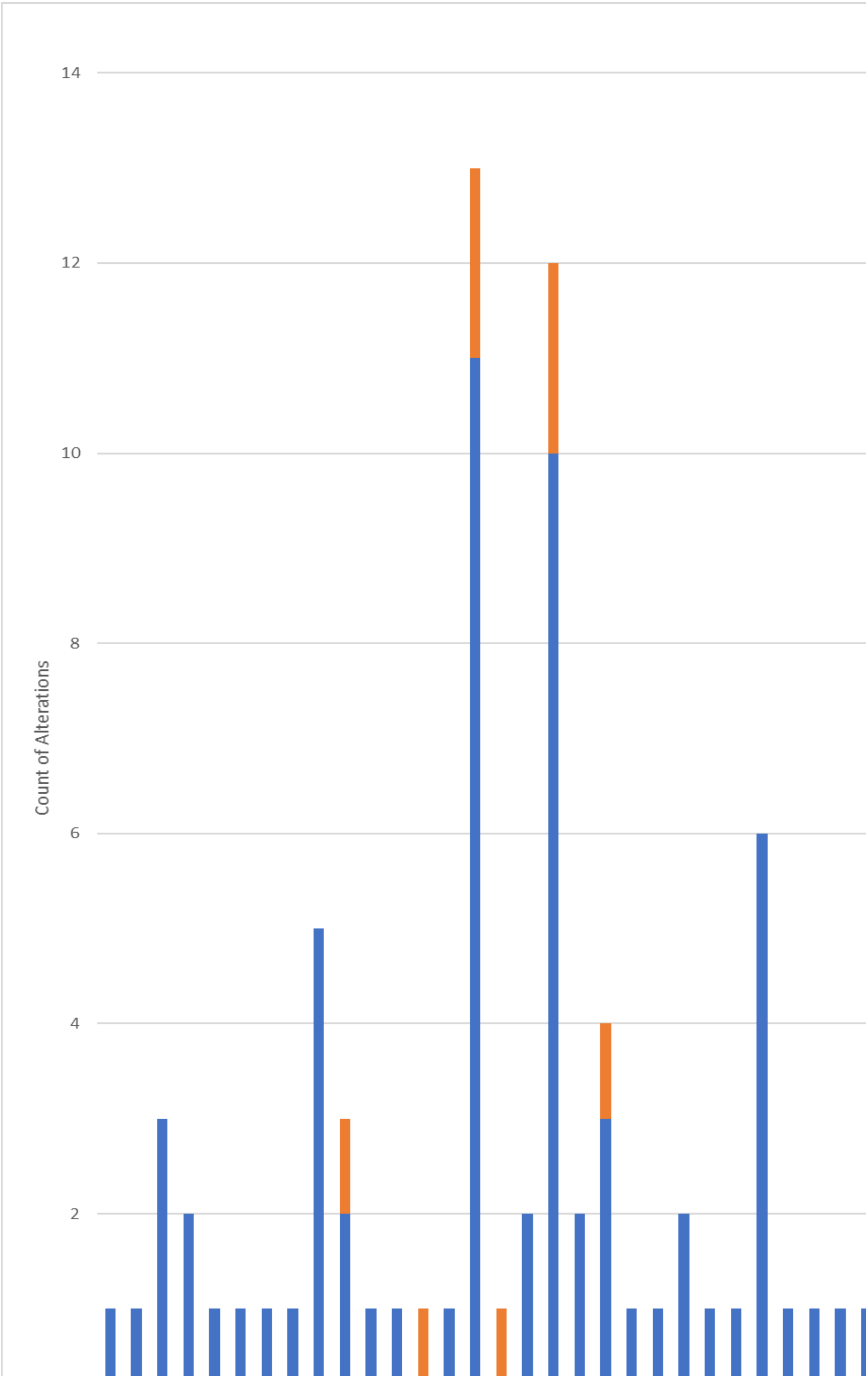
14
12
10
8
6
4
2
Count of Alterations

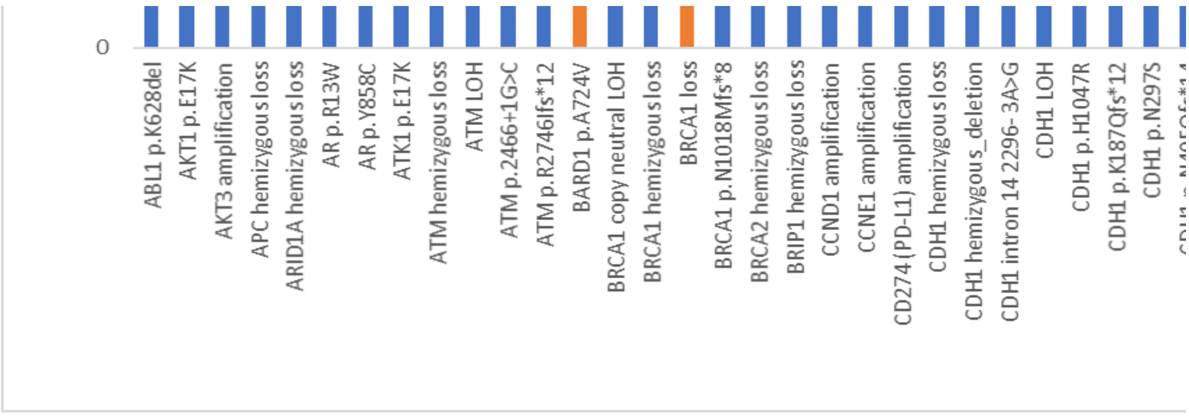

For Review Only

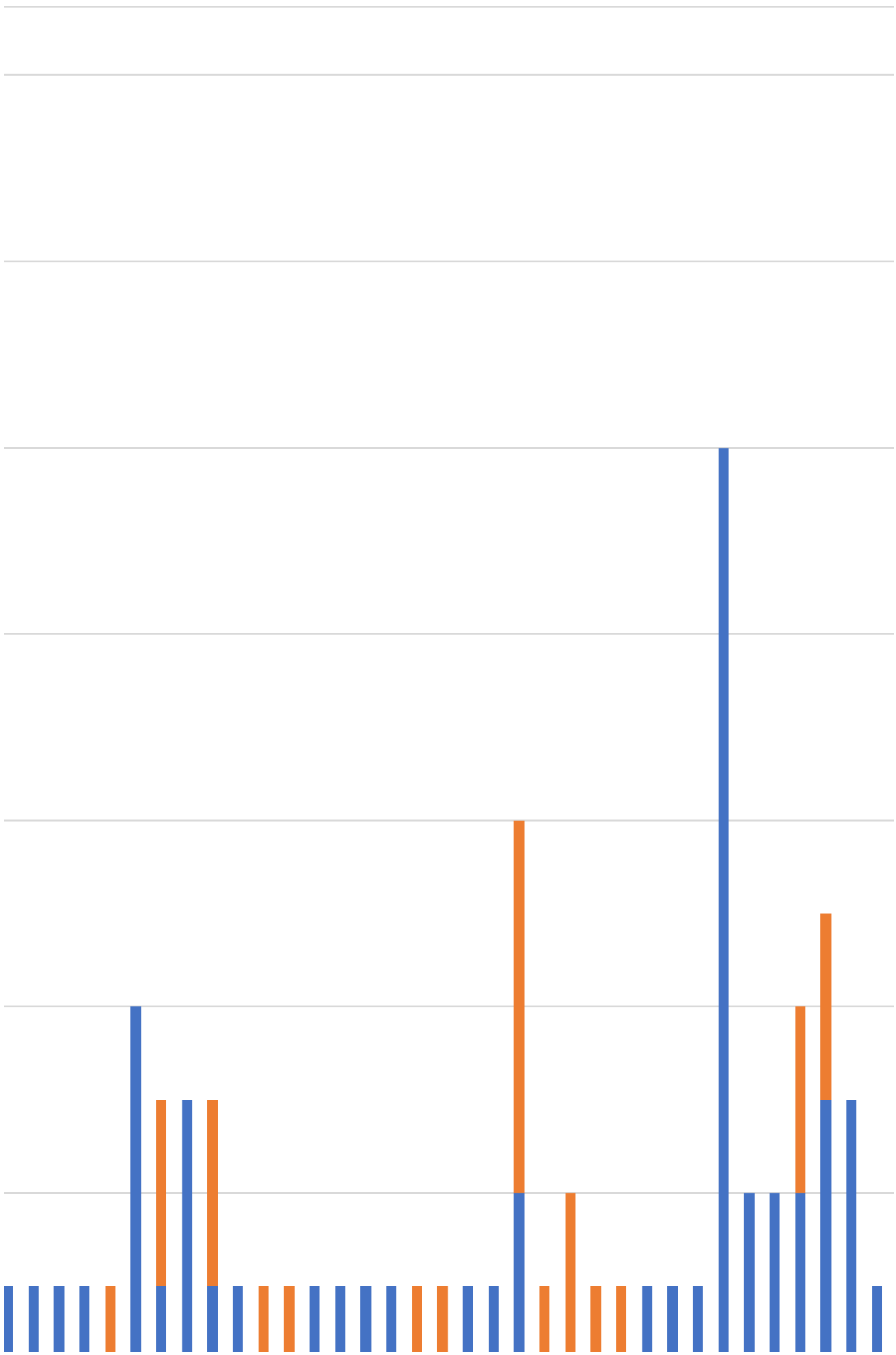

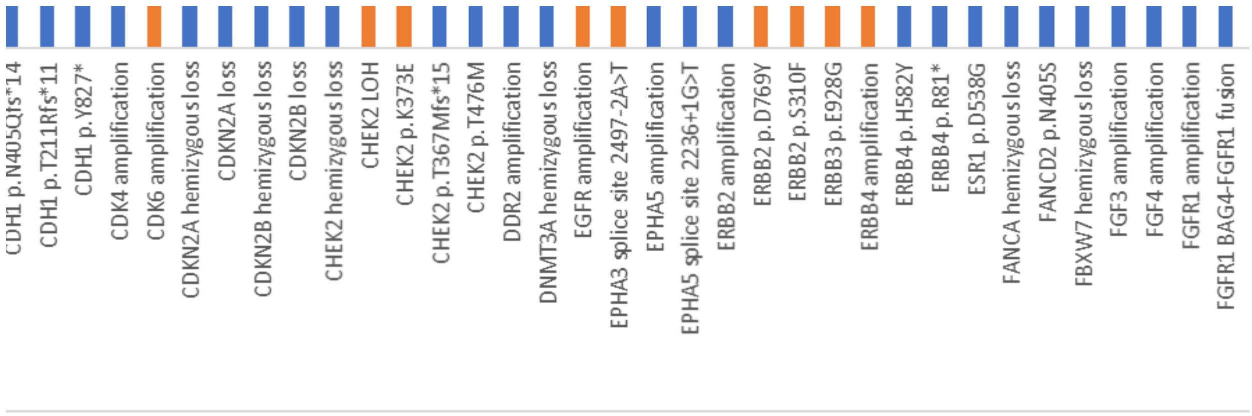

For Review Only

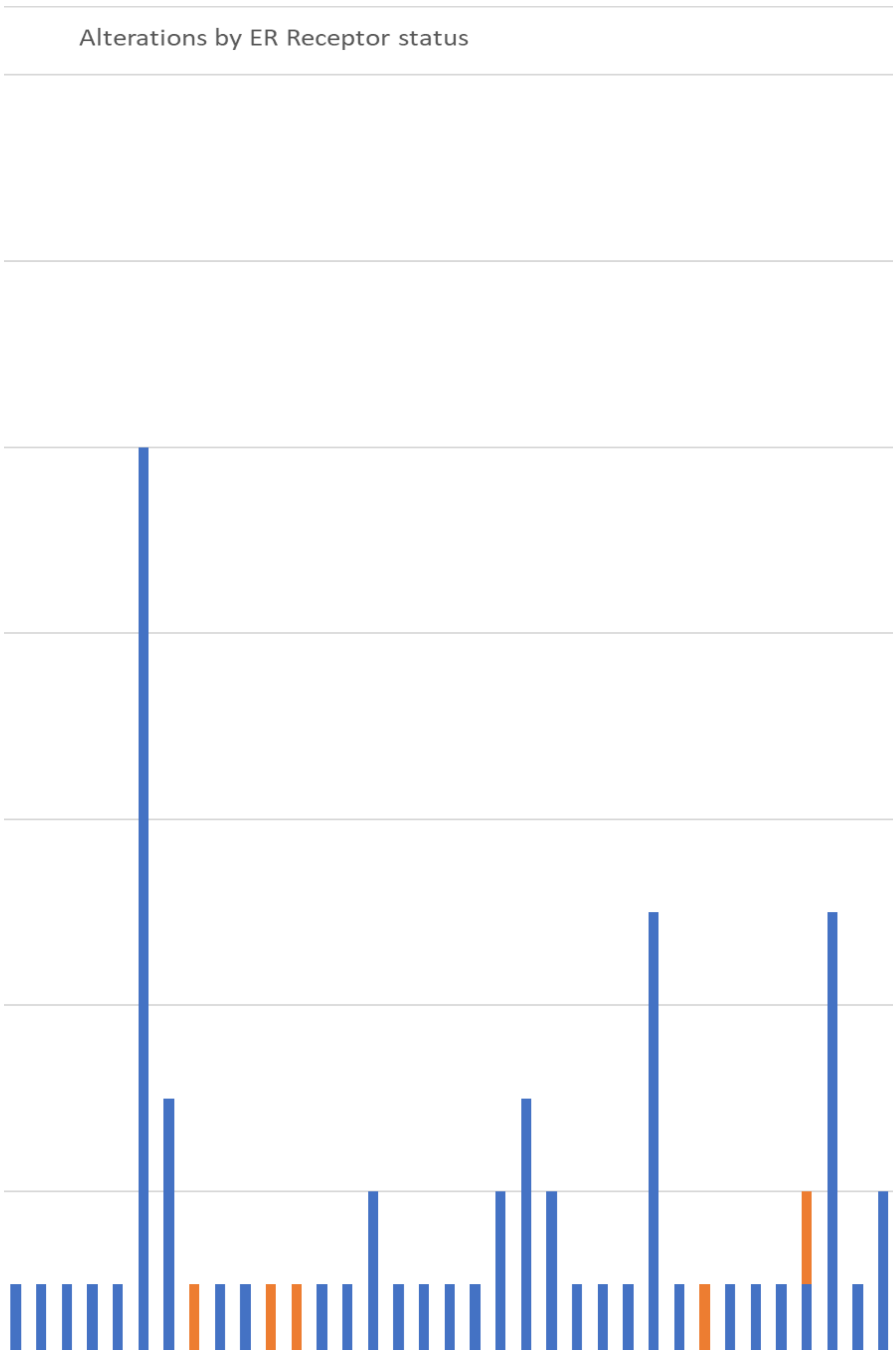
Alterations by ER Receptor status

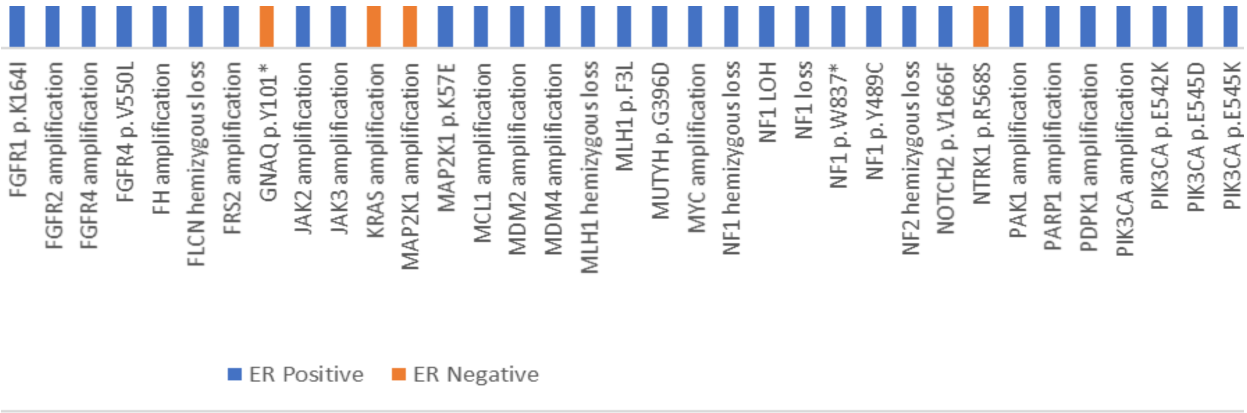

For Review Only

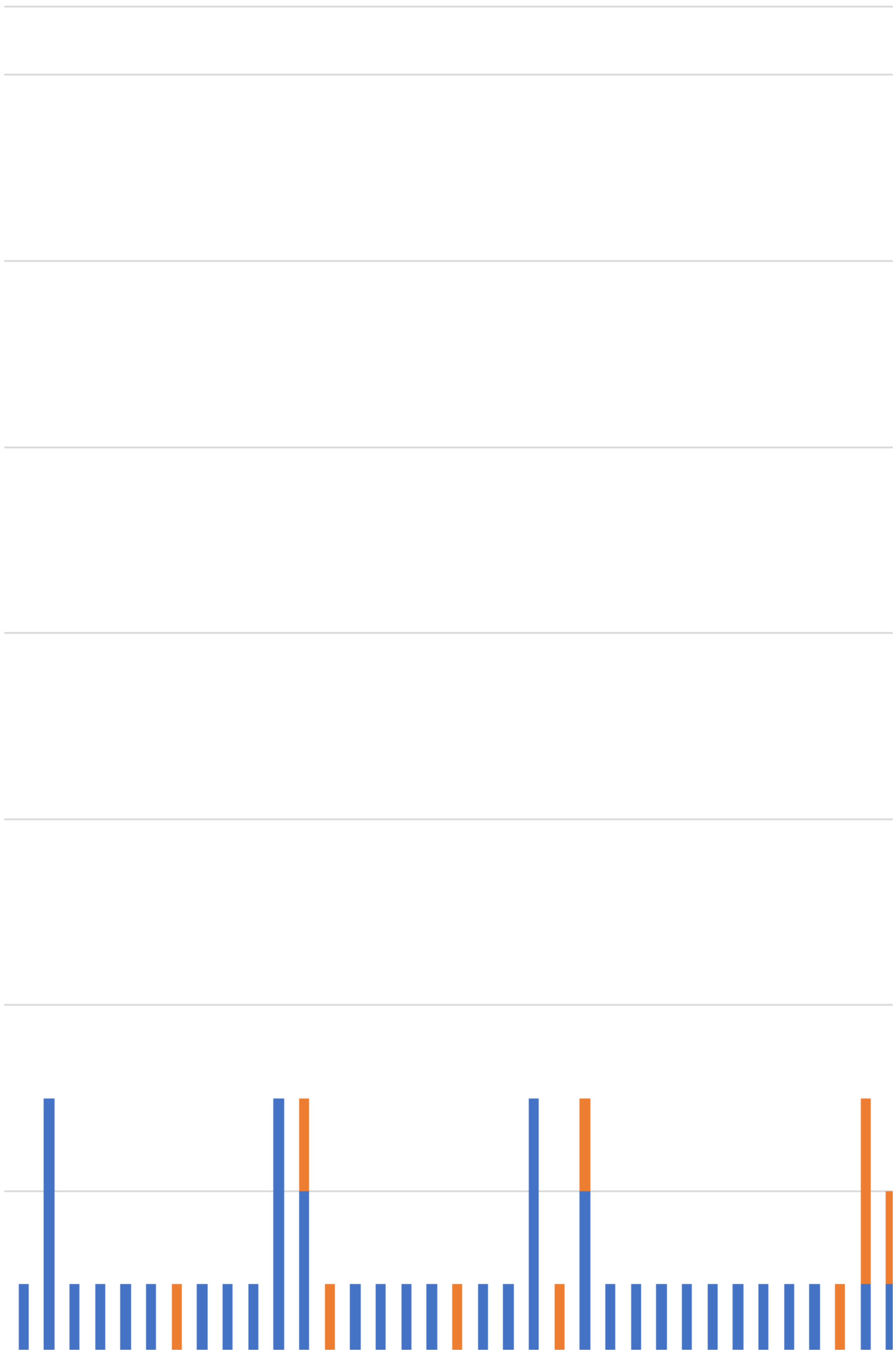

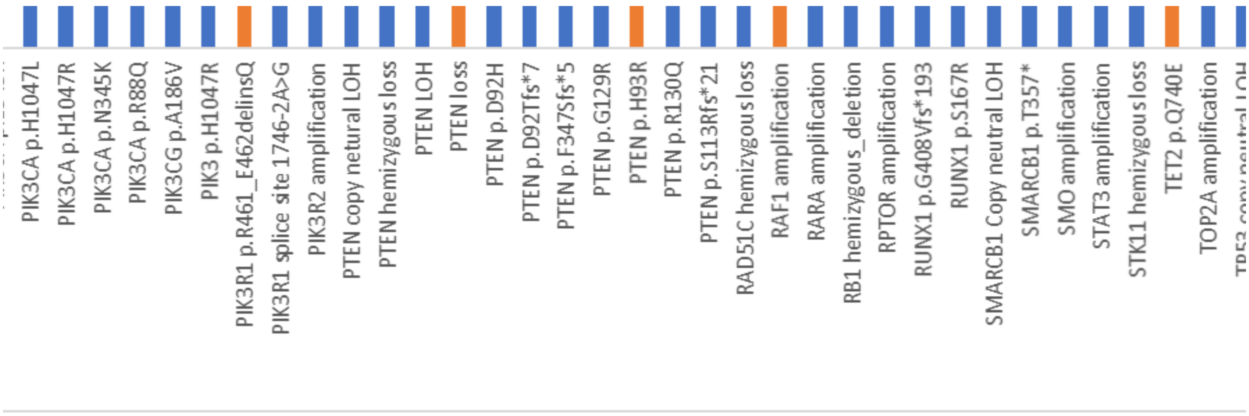

For Review Only

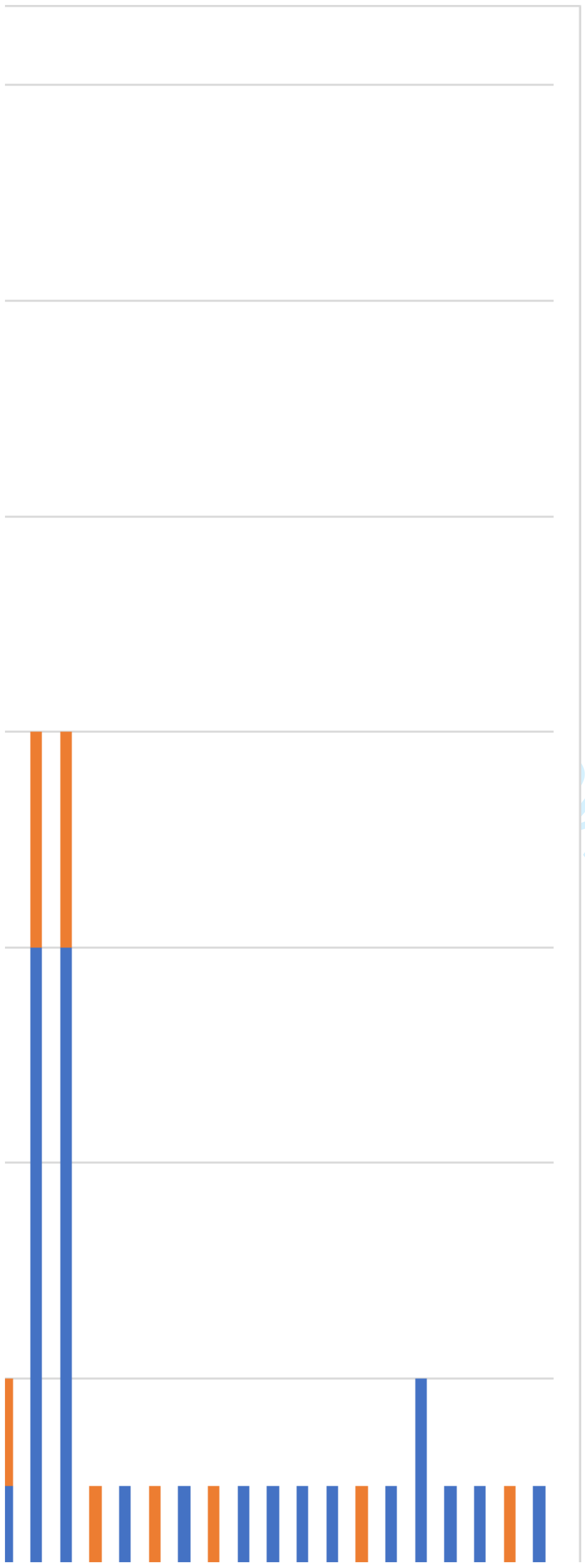

w Only

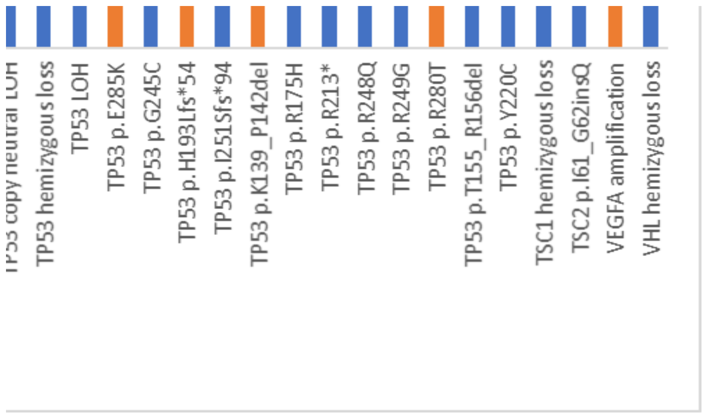

For Review Only

Supplement Table 2: Genetic Alterations Found and Available MTA

Gene	Alteration	Number of times observed[a]	Source
ABL1	p.K628del	1	Somatic
AKT1	p.E17K	2	Somatic
AKT3	amplification	3	Somatic
APC	hemizygous loss	2	Somatic
AR	p.R13W	1	Somatic
AR	p.Y858C	1	Somatic
ARID1A	hemizygous loss	1	Somatic
ATM	p.2466+1G>C	1	Germline
ATM	LOH	3	Somatic
ATM	p.R2746Ifs*12	1	Somatic
ATM	hemizygous loss	5	Somatic
BARD1	p.A724V	1	Somatic
CHEK2	p.T367Mfs*15	1	Germline
BRCA1	hemizygous loss	13	Somatic
BRCA1	loss	1	Somatic
BRCA1	copy neutral LOH	1	Somatic
BRCA2	hemizygous loss	12	Somatic
BRIP1	hemizygous loss	2	Somatic
CCND1	amplification	4	Somatic
CCNE1	amplification	1	Somatic
CD274 (PD-L1)	amplification	1	Somatic
CDH1	p.N297S	1	Somatic
CDH1	p.N405Qfs*14	1	Somatic
CDH1	LOH	6	Somatic
CDH1	p.Y827*	1	Somatic
CDH1	p.H1047R	1	Somatic
CDH1	p.K187Qfs*12	1	Somatic
CDH1	intron 14 2296- 3A>G	1	Somatic
CDH1	hemizygous_deletion	1	Somatic
CDH1	hemizygous loss	2	Somatic
CDH1	p.T211Rfs*11	1	Somatic
CDK4	amplification	1	Somatic
CDK6	amplification	1	Somatic
CDKN2A	loss	3	Somatic
CDKN2A	hemizygous loss	4	Somatic
CDKN2B	loss	3	Somatic
CDKN2B	hemizygous loss	3	Somatic
CHEK2	p.T476M	1	Germline
CHEK2	hemizygous loss	1	Somatic
CHEK2	p.K373E	1	Somatic
CHEK2	LOH	1	Somatic
DDR2	amplification	1	Somatic
DNMT3A	hemizygous loss	1	Somatic
EGFR	amplification	1	Somatic
EPHA3	splice site 2497-2A>T	1	Somatic
EPHA5	amplification	1	Somatic
EPHA5	splice site 2236+1G>T	1	Somatic

For Peer Review Only

ERBB2	amplification	6	Somatic
ERBB2	p.S310F	2	Somatic
ERBB2	p.D769Y	1	Somatic
ERBB3	p.E928G	1	Somatic
ERBB4	amplification	1	Somatic
ERBB4	p.H582Y	1	Somatic
ERBB4	p.R81*	1	Somatic
ESR1	p.D538G	1	Somatic
FANCA	hemizygous loss	10	Somatic
FANCD2	p.N405S	2	Somatic
FBXW7	hemizygous loss	2	Somatic
FGF3	amplification	4	Somatic
FGF4	amplification	5	Somatic
FGFR1	amplification	3	Somatic
FGFR1	BAG4-FGFR1 fusion	1	Somatic
FGFR1	p.K164I	1	Somatic
FGFR2	amplification	1	Somatic
FGFR4	p.V550L	1	Somatic
FGFR4	amplification	1	Somatic
FH	amplification	1	Somatic
FLCN	hemizygous loss	10	Somatic
FRS2	amplification	3	Somatic
GNAQ	p.Y101*	1	Somatic
JAK2	amplification	1	Somatic
JAK3	amplification	1	Somatic
KRAS	amplification	1	Somatic
MAP2K1	p.K57E	1	Somatic
MAP2K1	amplification	1	Somatic
MCL1	amplification	1	Somatic
MDM2	amplification	2	Somatic
MDM4	amplification	1	Somatic
MLH1	p.F3L	1	Somatic
MLH1	hemizygous loss	1	Somatic
MUTYH	p.G396D	1	Germline
MYC	amplification	2	Somatic
NF1	hemizygous loss	3	Somatic
NF1	LOH	2	Somatic
NF1	p.W837*	1	Somatic
NF1	p.Y489C	1	Somatic
NF1	loss	1	Somatic
NF2	hemizygous loss	5	Somatic
NOTCH2	p.V1666F	1	Somatic
NTRK1	p.R568S	1	Somatic
PAK1	amplification	1	Somatic
PARP1	amplification	1	Somatic
PDPK1	amplification	1	Somatic
PIK3	p.H1047R	1	Somatic
PIK3CA	p.E542K	5	Somatic
PIK3CA	p.H1047R	3	Somatic
PIK3CA	p.H1047L	1	Somatic
PIK3CA	p.E545K	2	Somatic
PIK3CA	p.E545D	1	Somatic

For Review Only

PIK3CA	p.N345K	1	Somatic
PIK3CA	amplification	2	Somatic
PIK3CA	p.R88Q	1	Somatic
PIK3CG	p.A186V	1	Somatic
PIK3R1	splice site 1746-2A>G	1	Somatic
PIK3R1	p.R461_E462delinsQ	1	Somatic
PIK3R2	amplification	1	Somatic
PTEN	hemizygous loss	3	Somatic
PTEN	LOH	3	Somatic
PTEN	p.S113Rfs*21	1	Somatic
PTEN	p.D92Tfs*7	1	Somatic
PTEN	loss	1	Somatic
PTEN	p.D92H	1	Somatic
PTEN	p.G129R	1	Somatic
PTEN	p.F347Sfs*5	1	Somatic
PTEN	p.R130Q	1	Somatic
PTEN	copy netural LOH	1	Somatic
PTEN	p.H93R	1	Somatic
RAD51C	hemizygous loss	3	Somatic
RAF1	amplification	1	Somatic
RARA	amplification	3	Somatic
RB1	hemizygous_deletion	1	Somatic
RPTOR	amplification	1	Somatic
RUNX1	p.G408Vfs*193	1	Somatic
RUNX1	p.S167R	1	Somatic
SMARCB1	p.T357*	1	Somatic
SMARCB1	Copy neutral LOH	1	Somatic
SMO	amplification	1	Somatic
STAT3	amplification	1	Somatic
STK11	hemizygous loss	1	Somatic
TET2	p.Q740E	1	Somatic
TOP2A	amplification	3	Somatic
TP53	p.Y220C	2	Somatic
TP53	p.G245C	1	Somatic
TP53	LOH	9	Somatic
TP53	p.E285K	1	Somatic
TP53	p.K139_P142del	1	Somatic
TP53	copy neutral LOH	1	Somatic
TP53	hemizygous loss	8	Somatic
TP53	p.R175H	1	Somatic
TP53	p.R280T	1	Somatic
TP53	p.R248Q	1	Somatic
TP53	p.I251Sfs*94	1	Somatic
TP53	p.T155_R156del	1	Somatic
TP53	p.R213*	1	Somatic
TP53	p.H193Lfs*54	1	Somatic
TP53	p.R249G	1	Somatic
TSC1	hemizygous loss	1	Somatic
TSC2	p.I61_G62insQ	1	Somatic
VEGFA	amplification	1	Somatic
VHL	hemizygous loss	1	Somatic

[a]Some alterations recurred in multiple patients.

For Review Only

[b]Not every drug was available to each patient and associated therapy is reported based on availal

LOH=loss of heterozygosity

For Review Only

FDA Approved Therapies for Breast Cancer	Reference
None	
Everolimus	PMID: 23215720
Everolimus	PMID: 23215720
None	
None	
None	
None	
None	
None	
None	
None	
None	
None	
None	
None	
Olaparib	PMID: 21424107, 21771338, 18971340, 18649131, 21482494
Olaparib	PMID: 21424107, 21771338, 18971340, 18649131, 21482494
None	
Palbociclib	PMID: 25524798, 26030518
None	
None	
None	
None	
None	
None	
None	
None	
None	
None	
None	
None	
Palbociclib	PMID: 25524798, 26030518
Palbociclib	PMID: 25524798, 26030519
Palbociclib	PMID: 25524798, 26030520
Palbociclib	PMID: 25524798, 26030521
Palbociclib	PMID: 25524798, 26030522
Palbociclib	PMID: 25524798, 26030523
None	
None	
None	
None	
None	
None	
None	
None	
None	
None	

For Peer Review Only

Trastuzumab, Ado-trast	PMID: 20728210
Trastuzumab,Adotrastu	PMID: 20728210
None	
Pertuzumab	
Lapatinib	PMID:16014882, 22285168, 23816960, 24263064, 19692680
None	
None	
None	
None	
None	
Everolimus	PMID: 23215720
None	
None	
None	
None	
None	
None	
None	
None	
None	
Everolimus	PMID: 23215720
None	
None	
None	
None	
None	
None	
None	
None	
None	
None	
None	
None	
None	
None	
Everolimus	PMID: 23215720
Everolimus	PMID: 23215721
Everolimus	PMID: 23215722
Everolimus	PMID: 23215723
Everolimus	PMID: 23215724
Everolimus, Lapatinib	PMID: 23215725
None	
None	
None	
None	
None	
Everolimus	PMID: 23215720
Everolimus	PMID: 23215721
Everolimus	PMID: 23215722
Everolimus	PMID: 23215723
Everolimus	PMID: 23215724
Everolimus	PMID: 23215725

For Review Only

Everolimus	PMID: 23215726
Everolimus	PMID: 23215727
Everolimus	PMID: 23215728
None	
Everolimus	PMID: 23215720
None	
None	
None	
Everolimus	PMID: 23215720
Everolimus	PMID: 23215721
Everolimus	PMID: 23215722
Everolimus	PMID: 23215723
Everolimus	PMID: 23215724
Everolimus	PMID: 23215725
Everolimus	PMID: 23215726
Everolimus	PMID: 23215727
Everolimus	PMID: 23215728
None	
None	
None	
None	
None	
Everolimus	PMID: 23215720
None	
None	
None	
None	
None	
None	
None	
None	
None	
None	
None	
None	
None	
None	
None	
None	
None	
None	
None	
None	
None	
None	
None	
None	
None	
None	
None	
Everolimus	PMID: 23215720

bility at the time of testing.

For Review Only

FDA Approved Therapies for Other Tumor Type	Clinical Trial Available
None	None
Everolimus,Temsirolimus	Yes
Everolimus,Temsirolimus	Yes
Celecoxib,Aspirin	Yes
None	None
None	None
None	Yes
Olaparib	yes
Olaparib,Rucaparib	Yes
None	Yes
Olaparib	yes
None	None
None	None
Olaparib, Niraparib,Rucaparib	Yes
None	Yes
None	Yes
Olaparib, Niraparib,Rucaparib	Yes
None	None
Palbociclib	Yes
None	None
Pembrolizumab, Atezolizumab, Nivolumab	Yes
None	None
None	None
None	None
None	None
None	None
None	None
None	None
None	None
None	None
None	None
None	Yes
None	Yes
None	yes
None	yes
None	Yes
None	Yes
None	None
None	None
None	None
None	None
Imatinib,Nilotinib,Dasatinib	Yes
Azacitidine,Decitabine	Yes
Afatinib, Gefitinib, Cetuximab, Erlotinib, Panitumumab, Necitumumab	Yes
None	None
None	None
None	None

For Review Only

Trastuzumab, Afatinib	Yes
Trastuzumab,Lapatinib,Afatinib	Yes
Ado-trastuzumab emtansine, Lapatinib, Pertuzumab, Trastuzumab, Afatinib	Yes
Lapatinib,Afatinib,Gefitinib	Yes
Gefitinib, Afatinib, Erlotinib	Yes
None	None
None	None
None	None
Olaparib	Yes
None	None
Everolimus,Temsirolimus	Yes
Pazopanib,Ponatinib,Regorafenib,Lenvatinib,Nintedanib	Yes
Pazopanib,Ponatinib,Regorafenib,Lenvatinib,Nintedanib	Yes
Lenvatinib, Ponatinib, Pazopanib, Nintedanib, Regorafenib	Yes
Lenvatinib, Regorafenib, Ponatinib, Pazopanib, Nintedanib	Yes
None	None
Lenvatinib,Regorafenib,Pazopanib,Ponatinib,Nintedanib	Yes
None	None
Lenvatinib, Ponatinib	Yes
None	None
Everolimus,Temsirolimus	Yes
None	None
None	None
Ruxolitinib	Yes
Ruxolitinib, Tofacitinib	Yes
Trametinib, Cobimetinib	Yes
Trametinib, Cobimetinib	Yes
Trametinib, Cobimetinib	Yes
None	None
None	Yes
None	None
None	None
Atezolizumab,Pembrolizumab,Nivolumab	Yes
None	None
None	Yes
Everolimus, Temsirolimus, Trametinib, Cobimetinib	yes
Everolimus,Temsirolimus,Trametinib,Cobimetinib	Yes
Everolimus, Temsirolimus, Trametinib, Cobimetinib	Yes
Everolimus,Temsirolimus,Trametinib,Cobimetinib	Yes
Everolimus,Temsirolimus,Trametinib,Cobimetinib	Yes
Everolimus,Lapatinib,Temsirolimus,Trametinib	Yes
None	None
None	None
None	None
Olaparib	Yes
None	None
Everolimus, Temsirolimus	Yes
Everolimus, Temsirolimus	Yes
Everolimus,Temsirolimus	Yes
Everolimus, Temsirolimus	Yes
Everolimus,Temsirolimus	Yes
Everolimus, Temsirolimus	Yes

For Review Only

Everolimus, Temsirolimus	Yes
Everolimus, Temsirolimus	Yes
Everolimus, Temsirolimus	yes
None	None
Everolimus, Temsirolimus	Yes
Temsirolimus, Everolimus	Yes
Everolimus, Temsirolimus	Yes
Everolimus,Temsirolimus	
Everolimus, Temsirolimus	Yes
Everolimus, Temsirolimus	Yes
Everolimus, Temsirolimus	Yes
Everolimus, Temsirolimus	Yes
Everolimus, Temsirolimus	yes
Everolimus,Temsirolimus	Yes
Everolimus,Temsirolimus	Yes
Everolimus,Temsirolimus	Yes
Everolimus,Temsirolimus	Yes
Everolimus,Temsirolimus	Yes
Olaparib, Niraparib,Rucaparib	Yes
Sorafenib,Cobimetinib,Trametinib,Regorafenib	Yes
Atra,Ato	No
None	None
Everolimus, Temsirolimus	Yes
None	None
None	None
None	None
None	Yes
Vismodegib, Sonidegib	
Ruxolitinib	Yes
Dasatinib, Everolimus, Temsirolimus, Bosutinib	Yes
None	None
None	None
None	Yes
None	yes
None	yes
None	yes
None	Yes
None	Yes
None	Yes
None	Yes
None	Yes
None	Yes
None	Yes
None	Yes
None	Yes
None	Yes
None	Yes
Everolimus,Temsirolimus	Yes
None	None
Ramucirumab, Sorafenib, Sunitinib, Bevacizumab, Axitinib, Lenvatinib, Pazopanib, Regoraf	Yes
Everolimus,Bevacizumab,Sunitinib,Temsirolimus,Vandetanib,Pazopanib,Sorafenib,Axitinib	Yes

For Review Only

For Review Only

Supplement Table 3: Patient Demo

DE-IDENTIFIED ID	Origine of Sample
381373	Primary
416170	Primary
375882	Primary
381163	Primary
384827	Primary
408655	Metastasis
411460	Metastasis
416144	Primary
487820	Primary
385187	Primary
413764	Primary
419398	Primary
374383	Primary
376490	Metastasis
381390	Metastasis
400589	Primary
404122	Metastasis
418122	Primary
435327	Primary
464520	Primary
370700	Metastasis
373384	Metastasis
432004	Primary
441866	Primary
482444	Primary
489490	Primary
419641	Primary
391993	Primary
508422	Primary
429264	Primary
429968	Primary
430972	Primary
469972	Primary

For Review Only

480099	Primary
487331	Primary
398306	Primary
411436	Primary
486602	Primary
425270	Primary
410276	Metastasis
441238	Primary
466056	Primary
391849	Primary

For Review Only

graphic Information

Patient Clinicial History and Pathologic Dia

Breast - type	Histology
ILC - grade 2-3	ILC
IDC - grade unknown	IDC
ILC - grade 2	ILC
IDC - grade 2	IDC
DCIS - grade 3	DCIS ONLY
Carcinoma, unspecified; bx of mets	Unknown Histology
IDC - grade unknown	IDC
IDC - grade 3; DCIS - grade 2	Mixed Histology
IDC, Neuroendocrine features - grade unknown	IDC
IDC - grade 3	IDC
IDC - grade 2	IDC
IDC - grade 3	IDC
IDC - grade 3	IDC
IDC - grade 2	IDC
IDC - grade 3	IDC
IDC - grade 2	IDC
IDC - grade 3	IDC
IDC - grade 3; DCIS - grade 3	Mixed Histology
IDC - grade 3	IDC
IDC - grade 2	IDC
IDC - grade 3	IDC
DCIS - grade 2, invasive, ductal and lobular features grade 2 out 3	Mixed Histology
IDC - grade unknown	IDC
ILC - grade unknown	ILC
ILC - grade 2	ILC
IDC - grade 2	IDC
IDC - grade 2	IDC
ILC - grade unknown	ILC
IDC - grade 1; DCIS - grade unknown	Mixed Histology
ILC - grade 2	ILC
ILC - grade 2	ILC
IDC - grade 2	IDC
IDC - grade unknown	IDC

For Review Only

IDC - grade 2; DCIS - grade 2	Mixed Histology
IDC - grade 3	IDC
DCIS - grade 3	DCIS ONLY
IDC - grade 2	IDC
IDC - grade 3	IDC
IDC - grade 2; IDC - grade 2	IDC
IDC - grade 3; DCIS - grade 3	Mixed Histology
IDC - grade 3	IDC
IDC - grade 3	IDC
IDC - grade unknown	IDC

For Review Only

agnosis		Somatic Alterations			
**Age at DX Group (%)**	**Breast Receptor Status**	**Somatic Alteration**	**Gene 1**	**Alteration 1**	**Gene 2**
60-69	pos/pos/neg	0			
50-59	pos/pos/pos	0			
50-59	pos/pos/neg	2	*CDH1*	p.Y827*	*CDH1*
40-49	pos/pos/neg	2	*FGF3*	amplification	*FGF4*
40-49	neg/neg/neg	2	*TP53*	p.E285K	*TP53*
70-79	pos/pos/neg	2	*ESR1*	p.D538G	*PIK3CA*
60-69	pos/pos/neg	2	*FGFR1*	amplification	*PIK3CA*
80 and over	pos/pos/neg	2	*PIK3CA*	p.E542K	*DNMT3A*
50-59	pos/pos/neg	2	*TP53*	hemizygous loss	*NF2*
60-69	neg/neg/neg	3	*ERBB2*	p.D769Y	*NTRK1*
50-59	pos/pos/neg	3	*PIK3R1*	splice site 1746-2A>G	*PTEN*
80 and over	pos/neg/neg	3	*BRCA1*	hemizygous loss	*PTEN*
60-69	pos/pos/neg	4	*AR*	p.R13W	*CDH1*
60-69	pos/pos/neg	4	*ATM*	p.R2746Ifs*12	*ATM*
70-79	pos/pos/neg	4	*AR*	p.Y858C	*PIK3CG*
60-69	pos/pos/neg	4	*PIK3CA*	p.H1047R	*CDH1*
60-69	neg/neg/neg	4	*BARD1*	p.A724V	*KRAS*
50-59	neg/neg/pos	4	*ERBB2*	amplification	*RARA*
60-69	neg/neg/neg	4	*PIK3R1*	p.R461_E462delinsQ	*FGF3*
60-69	pos/pos/neg	4	*PIK3CA*	p.H1047R	*FANCA*
30-39	pos/pos/neg	6	*AKT1*	p.E17K	*FRS2*
70-79	pos/pos/neg	6	*PIK3CA*	p.E545K	*AKT3*
40-49	pos/neg/neg	6	*FGFR1*	BAG4-FGFR1 fusion	*FGFR1*
40-49	pos/pos/neg	6	*PIK3CA*	p.E542K	*PIK3CA*
60-69	pos/pos/neg	6	*AKT1*	p.E17K	*VHL*
80 and over	neg/neg/neg	6	*ERBB3*	p.E928G	*ERBB2*
40-49	neg/neg/pos	7	*BRCA1*	hemizygous loss	*CDKN2A*
80 and over	pos/pos/neg	8	*BRCA2*	hemizygous loss	*CDH1*
40-49	pos/pos/pos	8	*BRCA1*	hemizygous loss	*BRCA2*
80 and over	pos/neg/neg	9	*BRCA1*	hemizygous loss	*CDH1*
50-59	pos/pos/neg	9	*AKT3*	amplification	*CDH1*
70-79	pos/pos/neg	9	*ATM*	hemizygous loss	*BRCA1*
50-59	pos/pos/neg	9	*PIK3CA*	p.E545K	*AKT3*

For Review Only

50-59	pos/pos/pos	9	*CDKN2A*	loss	*CDKN2B*
70-79	pos/neg/pos	9	*ERBB2*	amplification	*CCND1*
80 and over	neg/pos/pos	10	*BRCA1*	loss	*CDKN2A*
50-59	pos/neg/NOS	10	*CDKN2A*	hemizygous loss	*CDKN2B*
80 and over	pos/pos/neg	13	*CDK4*	amplification	*CDKN2A*
70-79	pos/neg/neg; pos/pos/neg	14	*CCND1*	amplification	*PIK3CA*
60-69	neg/neg/pos	14	*ATM*	LOH	*BRCA1*
60-69	pos/pos/neg	16	*BRCA1*	copy neutral LOH	*BRCA2*
50-59	pos/neg/neg	16	*PTEN*	p.G129R	*PTEN*
40-49	pos/pos/pos	17	*ERBB2*	amplification	*PDPK1*

For Review Only

**Alteration 2**	**Gene 3**	**Alteration 3**	**Gene 4**	**Alteration 4**
LOH				
amplification				
LOH				
p.E545D				
p.N345K				
hemizygous loss				
hemizygous loss				
p.R568S	*TET2*	p.Q740E		
p.S113Rfs*21	*PTEN*	LOH		
p.D92Tfs*7	*TP53*	p.R175H		
p.N297S	*PIK3CA*	p.H1047L	*TP53*	p.Y220C
LOH	*PIK3CA*	p.E542K	*RUNX1*	p.S167R
p.A186V	*TP53*	p.G245C	*TP53*	LOH
hemizygous_deletion	*RB1*	hemizygous_deletion	*FANCD2*	p.N405S
amplification	*TP53*	p.K139_P142del	*TP53*	copy neutral LOH
amplification	*TOP2A*	amplification	*TP53*	hemizygous loss
amplification	*FGF4*	amplification	*ERBB2*	p.S310F
hemizygous loss	*CDH1*	hemizygous loss	*NOTCH2*	p.V1666F
amplification	*MAP2K1*	p.K57E	*MDM2*	amplification
amplification	*FANCA*	hemizygous loss	*FLCN*	hemizygous loss
amplification	*NF1*	p.W837*	*NF1*	LOH
amplification	*FGFR1*	amplification	*BRCA2*	hemizygous loss
hemizygous loss	*BRCA1*	hemizygous loss	*BRCA2*	hemizygous loss
p.S310F	*PTEN*	p.H93R	*PTEN*	LOH
loss	*CDKN2B*	loss	*ERBB2*	amplification
intron 14 2296- 3A>G	*CDH1*	LOH	*CHEK2*	hemizygous loss
hemizygous loss	*RAD51C*	hemizygous loss	*TP53*	p.Y220C
p.H1047R	*CDH1*	LOH	*FANCA*	hemizygous loss
p.K187Qfs*12	*CDH1*	LOH	*CDKN2A*	hemizygous loss
hemizygous loss	*CCND1*	amplification	*CDKN2A*	hemizygous loss
amplification	*DDR2*	amplification	*BRCA1*	hemizygous loss

For Review Only

loss	*PTEN*	p.R130Q	*PTEN*	copy netural LOH
amplification	*PIK3CA*	p.E542K	*PTEN*	hemizygous loss
loss	*CDKN2B*	loss	*EGFR*	amplification
hemizygous loss	*PTEN*	hemizygous loss	*NF2*	hemizygous loss
hemizygous loss	*CDKN2B*	hemizygous loss	*PTEN*	hemizygous loss
p.R88Q	*PTEN*	p.D92H	*PTEN*	LOH
hemizygous loss	*BRCA2*	hemizygous loss	*CCND1*	amplification
hemizygous loss	*CCNE1*	amplification	*EPHA5*	splice site 2236+1G>T
p.F347Sfs*5	*FGFR2*	amplification	*FBXW7*	hemizygous loss
amplification	*FGFR4*	amplification	*JAK2*	amplification

For Review Only

Gene 5	Alteration 5	Gene 6	Alteration 6	Gene 7	Alteration 7
MYC	amplification	*RUNX1*	p.G408Vfs*193		
CDH1	p.N405Qfs*14	*CDH1*	LOH		
BRCA2	hemizygous loss	*FLCN*	hemizygous loss		
TP53	p.R248Q	*TP53*	LOH		
CDH1	p.T211Rfs*11	*CDH1*	LOH		
BRCA2	hemizygous loss	*TP53*	hemizygous loss		
PIK3CA	amplification	*PTEN*	loss	*TP53*	p.R280T
FANCA	hemizygous loss	*FANCD2*	p.N405S	*NF2*	hemizygous loss
TP53	LOH	*FGFR1*	p.K164I	*ABL1*	p.K628del
FLCN	hemizygous loss	*NF1*	hemizygous loss	*PIK3CA*	p.H1047R
CDKN2B	hemizygous loss	*FANCA*	hemizygous loss	*NF2*	hemizygous loss
FANCA	hemizygous loss	*NF1*	hemizygous loss	*NF2*	hemizygous loss
PARP1	amplification	*MDM4*	amplification	*FH*	amplification

For Review Only

FBXW7	hemizygous loss	*NF1*	loss	*BRCA1*	hemizygous loss
FGF4	amplification	*FLCN*	hemizygous loss	*TP53*	p.R249G
EPHA3	splice site 2497-2A>T	*ERBB2*	amplification	*ERBB4*	amplification
BRCA2	hemizygous loss	*ATM*	LOH	*FANCA*	hemizygous loss
FRS2	amplification	*NF1*	hemizygous loss	*BRCA1*	hemizygous loss
FGF3	amplification	*FGF4*	amplification	*BRCA2*	hemizygous loss
CDK6	amplification	*CHEK2*	p.K373E	*CHEK2*	LOH
FLCN	hemizygous loss	*JAK3*	amplification	*MLH1*	p.F3L
NF1	p.Y489C	*NF1*	LOH	*BRCA1*	hemizygous loss
STAT3	amplification	*SMARCB1*	p.T357*	*SMARCB1*	Copy neutral LOH

For Review Only

Gene 8	Alteration 8	Gene 9	Alteration 9	Gene 10	Alteration 10	Gene 11
PIK3	p.H1047R					
BRIP1	hemizygous loss					
RAD51C	hemizygous loss	*TP53*	hemizygous loss			
PIK3CA	p.E542K	*TP53*	hemizygous loss			
PAK1	amplification	*TP53*	hemizygous loss			
MCL1	amplification	*BRIP1*	hemizygous loss			

For Review Only

BRCA2	hemizygous loss	*APC*	hemizygous loss			
TP53	LOH	*RARA*	amplification			
MAP2K1	amplification	*TOP2A*	amplification	*VEGFA*	amplification	
FLCN	hemizygous loss	*TP53*	p.T155_R156del	*TP53*	LOH	
ATM	hemizygous loss	*APC*	hemizygous loss	*FLCN*	hemizygous loss	*TSC1*
ATM	hemizygous loss	*FANCA*	hemizygous loss	*FLCN*	hemizygous loss	*EPHA5*
ERBB2	amplification	*FGF3*	amplification	*FGF4*	amplification	*GNAQ*
PIK3R2	amplification	*STK11*	hemizygous loss	*TP53*	p.I251Sfs*94	*TP53*
BRCA2	hemizygous loss	*ATM*	hemizygous loss	*FANCA*	hemizygous loss	*RAD51C*
FRS2	amplification	*BRCA1*	hemizygous loss	*ATM*	hemizygous loss	*CD274 (PD-L.*

For Review Only

Alteration 11	Gene 12	Alteration 12	Gene 13	Alteration 13	Gene 14

For Review Only

hemizygous loss	*TP53*	hemizygous loss	*CDH1*	hemizygous loss	
amplification	*FGFR4*	p.V550L	*ERBB4*	p.H582Y	*ERBB4*
p.Y101*	*RAF1*	amplification	*TP53*	p.H193Lfs*54	*TP53*
copy neutral LOH	*TSC2*	p.I61_G62insQ	*BRCA1*	hemizygous loss	*FANCA*
hemizygous loss	*MLH1*	hemizygous loss	*ARID1A*	hemizygous loss	*FLCN*
amplification	*MDM2*	amplification	*TP53*	hemizygous loss	*MYC*

For Review Only

Alteration 14	Gene 15	Alteration 15	Gene 16	Alteration 16	Gene 17

For Review Only

p.R81*					
LOH					
hemizygous loss	*FLCN*	hemizygous loss	*RPTOR*	amplification	
hemizygous loss	*TP53*	p.R213*	*TP53*	LOH	
amplification	*RARA*	amplification	*SMO*	amplification	*TOP2A*

For Review Only

	Germline Alterations			
Alteration 17	Germline Alteration Counts	Gene	Alteration	Germnline Classification
	0			
	0			
	0			
	0			
	0			
	0			
	0			
	0			
	0			
	0			
	0			
	0			
	1	*CHEK2*	p.T476M	VLP
	0			
	0			
	0			
	0			
	0			
	0			
	0			
	0			
	0			
	0			
	0			
	0			
	0			
	0			
	0			
	0			
	0			
	0			
	0			
	0			

For Review Only

	0			
	0			
	0			
	1	*ATM*	p.2466+1G>C	VLP
	1	*CHEK2*	p.T367Mfs*15	Mutation
	1	*MUTYH*	p.G396D	Mutation
	0			
	1	*BRCA1*	p.N1018Mfs*8	Mutation
	0			
amplification	0			

For Review Only
